# BCCIP is required for nucleolar recruitment of eIF6 and 12S pre-rRNA production during 60S ribosome biogenesis

**DOI:** 10.1093/nar/gkaa1114

**Published:** 2020-11-27

**Authors:** Caiyong Ye, Bochao Liu, Huimei Lu, Jingmei Liu, Arnold B Rabson, Estela Jacinto, Dimitri G Pestov, Zhiyuan Shen

**Affiliations:** Rutgers Cancer Institute of New Jersey, Department of Radiation Oncology, Rutgers Robert Wood Johnson Medical School, 195 Little Albany Street, New Brunswick, NJ 08901, USA; Rutgers Cancer Institute of New Jersey, Department of Radiation Oncology, Rutgers Robert Wood Johnson Medical School, 195 Little Albany Street, New Brunswick, NJ 08901, USA; Rutgers Cancer Institute of New Jersey, Department of Radiation Oncology, Rutgers Robert Wood Johnson Medical School, 195 Little Albany Street, New Brunswick, NJ 08901, USA; Rutgers Cancer Institute of New Jersey, Department of Radiation Oncology, Rutgers Robert Wood Johnson Medical School, 195 Little Albany Street, New Brunswick, NJ 08901, USA; Department of Pharmacology, and The Child Health Institute of New Jersey, Rutgers Robert Wood Johnson Medical School, New Brunswick, NJ, USA; Department of Biochemistry and Molecular Biology, Rutgers Robert Wood Johnson Medical School, Piscataway, NJ, USA; Department of Cell Biology and Neuroscience, Rowan University School of Osteopathic Medicine, Stratford, NJ, USA; Rutgers Cancer Institute of New Jersey, Department of Radiation Oncology, Rutgers Robert Wood Johnson Medical School, 195 Little Albany Street, New Brunswick, NJ 08901, USA

## Abstract

Ribosome biogenesis is a fundamental process required for cell proliferation. Although evolutionally conserved, the mammalian ribosome assembly system is more complex than in yeasts. BCCIP was originally identified as a BRCA2 and p21 interacting protein. A partial loss of *BCCIP* function was sufficient to trigger genomic instability and tumorigenesis. However, a complete deletion of *BCCIP* arrested cell growth and was lethal in mice. Here, we report that a fraction of mammalian BCCIP localizes in the nucleolus and regulates 60S ribosome biogenesis. Both abrogation of BCCIP nucleolar localization and impaired BCCIP–eIF6 interaction can compromise eIF6 recruitment to the nucleolus and 60S ribosome biogenesis. BCCIP is vital for a pre-rRNA processing step that produces 12S pre-rRNA, a precursor to the 5.8S rRNA. However, a heterozygous *Bccip* loss was insufficient to impair 60S biogenesis in mouse embryo fibroblasts, but a profound reduction of BCCIP was required to abrogate its function in 60S biogenesis. These results suggest that BCCIP is a critical factor for mammalian pre-rRNA processing and 60S generation and offer an explanation as to why a subtle dysfunction of BCCIP can be tumorigenic but a complete depletion of BCCIP is lethal.

## INTRODUCTION

Ribosomes are responsible for translating mRNAs into proteins. In eukaryotes, the ribosome is composed of the 40S and 60S subunits, which are assembled from about 80 ribosomal proteins and four distinct rRNAs (1). Ribosome biogenesis is a very complex process, which is fundamentally essential for cell viability and growth. In eukaryotic cells, ribosome assembly starts in the nucleolus followed by the export of nascent ribosomal subunits to the cytoplasm for final maturation. Ribosomal protein genes are transcribed by RNA polymerase II. Ribosomal proteins are synthesized in the cytoplasm, and imported into the nucleus for assembly with rRNAs, which are transcribed by RNA polymerases I and III. In addition to ribosomal proteins and rRNAs, about 200 assembly factors and 77 small nucleolar RNAs (snoRNAs) in yeast, and >500 assembly factors and 300 snoRNAs in higher eukaryotes have been found to participate in ribosome biogenesis (1). A significant number of mammalian ribosome assembly factors have functions distinct from their yeast homologues (2).

Although ribosome assembly factors are not physically part of the core ribosomes, they are critical for the generation of new ribosomes in cells. One such protein is the eukaryotic translation initiation factor 6 (eIF6), conserved from yeast to mammals (3). eIF6 has dual functions in ribosome biogenesis in the nucleolus and protein translation in the cytoplasm (4). In the nucleolus, the *Saccharomyces cerevisiae* Tif6 protein (the orthologue of mammalian eIF6) is required for the 60S ribosomal subunit biogenesis (5). Down-regulation of eIF6 in human cells reduced several rRNA precursors, especially 12S pre-rRNA, which is the precursor of the mature 5.8S rRNA component of 60S subunits (6–8). In the cytoplasm, eIF6 is disassociated from the 60S ribosomal subunit, before the 60S subunit binds with a 40S subunit to form an 80S ribosome (9). Due to its roles in ribosome biogenesis and in the regulation of translation, eIF6 is over-expressed in multiple types of cancer (8,10–15), and its over-expression is often associated with increased tumor aggressiveness (8,16,17). Deficiency of eIF6 influences the processing of rRNAs (8), however the molecular mechanisms of how eIF6 participates in ribosome assembly are not fully understood. Based on the cryo-electron microscopy (cryo-EM) structure of the 60S pre-ribosome in yeast (18), eIF6 directly interacts with ribosomal protein L23 (RPL23, uL14). Consistently, the same interaction between eIF6 and RPL23 has also been reported in the *Tetrahymena thermophila* 60S subunit crystal structure (19). This interaction is required for eIF6 recruitment to the pre-60S ribosome, and depletion of RPL23 reduced Tif6 recruitment to pre-ribosomes in yeast (20).

BCCIP was initially identified as a BRCA2 and p21 interacting protein, evolutionarily conserved from yeasts to mammals (4,21). BCCIP plays complex roles in cell proliferation and tumorigenesis. On the one hand, a partial *BCCIP* knockdown is sufficient to impair DNA damage repair, cell cycle regulation, mitotic spindle dynamics, and genomic stability (22–30). Mosaic and heterozygous Bccip deletions have been shown to cause chronic inflammation in mice and to lead to B-lymphoma and liver cancer (31). A transient *Bccip* down-regulation is not only sufficient, but also necessary for medulloblastoma development in mice (32). *BCCIP* down-regulation with normal p53 is associated with a poor outcome of laryngeal cancer (33). Thus, a partial BCCIP deficiency is a risk factor for tumorigenesis. On the other hand, a major or complete loss of BCCIP is detrimental to cellular proliferation. Mouse *Bccip* is essential for embryonic development (24,31), and induction of homozygous deletion of *Bccip* in adult mice resulted in acute death due to proliferation arrest in intestinal crypts (34).

Using a network guided computational approach, the yeast homologue of mammalian *BCCIP* gene, *Bcp1*, was suggested to be required for ribosomal biogenesis (35). A yeast temperature sensitive Bcp1 mutant exhibited deficits in 60S biogenesis (36). However, even though mammalian eIF6 can be co-precipitated with BCCIP (37), the yeast Bcp1 does not co-precipitate with Tif6 (36), and no defects in ribosome biogenesis were observed after *BCCIP* knockdown in HeLa cells (37). Thus, the specific contribution of mammalian *BCCIP* to ribosome biogenesis and its role in cell fitness remain to be addressed. In this study, we found that induced depletion of BCCIP in mouse and human cells caused an abrogation of 60S ribosome subunit biogenesis. We further showed that a fraction of human and mouse BCCIP localize in the nucleolus in an RNA- and DNA-dependent manner. BCCIP is required for the nucleolar recruitment of eIF6 and the generation of 12S pre-rRNA. Our results firmly establish mammalian BCCIP as a critical factor for 60S ribosomal subunit biogenesis.

## MATERIALS AND METHODS

### Cell lines and culture

HEK293, 293T, NIH3T3 and U2OS cells were cultured in Dulbecco's Modified Eagle's Medium, HT1080 cells were cultured in α-Dulbecco's modified Eagle’s medium. All cell culture media were supplemented with 10% fetal bovine serum, 20 mM glutamine and 1% penicillin–streptomycin. Mouse embryonic fibroblasts (MEFs) were isolated from *Bccip^flox/flox^;Rosa26-CreERT2* mice (34) and immortalized with the standard three-to-three (3T3) protocol (38), and routinely maintained in D-Dulbecco's modified Eagle's medium.

### Plasmid vectors and production of retroviruses and lentivirus

The cDNA of mouse *Bccip* (*mBccip*) was cloned into the pFLAG-CMV-2 vector (Addgene E7398) for transient expression. The *mBccip* mutants were generated with a site-directed mutagenesis Kit (E0554S, NEB). For transgene expression, cells were seeded overnight and were transfected at 30% confluence (100 mm dish) with 12 μg plasmid DNA using the polyethylenimine (PEI, Polysciences, 23966) according to the manufacturer's instructions. Four hours after transfection, the medium was changed to fresh medium, and cells were processed for specific assays as specified in respective experiments, such as immunofluorescence staining, immunoprecipitation, and western blots.

The cDNA was also cloned into the pLXSP vector and used to produce retrovirus as previously described (30). To enable stable expression of cDNA transgenes, cells were infected with retroviruses by three cycles of 8-h infection and 16-h of incubation with fresh medium, and then subjected to 1 μg/ml puromycin (Sigma) selection. Positive single and mixed clones were obtained, and the population was expanded to provide stable cell lines.

To construct inducible *BCCIP* knockdown, previously described *BCCIP* shRNA templates (4,30) were cloned into the pLKO-Neomycin Lenti-viral vector through the AgeI and EcoRI sites, resulting in pLKO-shBCCIPα-Neomycin, pLKO-shBCCIPβ-Neomycin, and pLKO-shBCCIPαβ-Neomycin. In the pLKO constructs, the BCCIP shRNA expression is under the control of the TetR site, and the shRNA were designed to target the sequence of 5′AACATCTCGGCACCTAGTA (shBCCIPα) in the 3′UTR of the BCCIPα, the sequence of 5′AACTCAGACTTTATTCAGA (shBCCIPβ) in 3′-UTR of the BCCIPβ, and the sequence of 5′GGCCTTCTCCTAAGTGAAA (shBCCIPαβ) in the common coding region 529–547 of BCCIPα and BCCIPβ mRNAs. To generate lentiviruses, 293T cells were seeded at 70% confluence for one day, cells were co-transfected with 6 μg pLKO-shBCCIP-Neomycin, 3 μg psPAX2 (Addgene, Cambridge, MA, USA, #12260), and 3 μg pMD2G (Addgene #12259). At 72 hours post the transfection, virus-containing supernatant was collected, filtered through a 0.45 μM nylon mesh and adjusted to 8 μg/ml polybrene (Sigma 107689). Target cells (HT1080, and U2OS) were incubated with viral supernatant overnight. Eighteen hours later, the supernatant was aspirated, and the cells were allowed to recover overnight, and then selected in Neomycin (Sigma, 800 μg/ml) for 72 h. The pLKO-Neomycin vector backbone was used as negative control. The oligonucleotide of 5′-ACACCCAAGGAGAAGAAGGC CAAGACCTCCAAGAAGAAGAAGCGCTCCAAGGCCAAGGCG-3′ that codes for TPKEKKAKTSKKKKRSKAKA was fused with GFP or GFP-eIF6 in the retroviral pLSXP vector, resulting in vectors that express NoLS-GFP and NoLS-GFP-eIF6.

### Exogenous expression of RNAi-resistant *BCCIP*

Because the shBCCIPαβ expressed from the lentivirus vectors was designed to target a shared coding region of the *BCCIPα* and *BCCIPβ* mRNAs at the following site: GGCCUUCUCCUAAGUGAAA, RNAi-resistant cDNAs were created by mutating four nucleotides in the shRNA-coding cDNA to 5′-GGGCTTCTGCTCAGCGAAA-3′. This produced RNAi-resistant BCCIP cDNA constructs, designated BCCIPα-M4 and BCCIPβ-M4, which were used for exogenous BCCIP expression in cells that express shBCCIPαβ.

### Antibodies and Western blotting

The rabbit anti-BCCIP BR5 and S1472–2 antibodies were made using recombinant BCCIP protein as antigens, and were previously characterized (24,30,32,39). Commercial antibodies used included antibodies against: p53 (sc-1801, Santa Cruz), β-tubulin (Sigma T8328, 1:1000), GAPDH (Sigma, 32233), GFP (Santa Cruz Biotechnology, Inc. sc-8334 1:1000), Flag (Cell Signaling Technology 1:500 #2368), RPL23 (BETHYL, A305–010A), eIF6 (Santa Cruz Biotechnology, sc-390432), eIF6 (Santa Cruz Biotechnology, sc-70270), B23 (Santa Cruz Biotechnology, FC-8791), nucleostemin (Santa Cruz Biotechnology, sc-166460), and p53 (Santa Cruz Biotechnology, FL-393), GAPDH (Santa Cruz Biotechnology, sc-32233), RPA194 (Santa Cruz Biotechnology, sc-48385), Fibrillarin (Santa Cruz Biotechnology, sc-374022), RPS3 (Santa Cruz Biotechnology, sc-376008), RPL22 (Santa Cruz Biotechnology, sc-373993), RPL23a(Santa Cruz Biotechnology, sc-517097), Nucleolin (Santa Cruz Biotechnology, sc-8031 FITC).

To perform Western blots, cells were lysed in RIPA buffer (50 mM Tris HCl, pH 7.4, with 150 mM NaCl, 1 mM EDTA, and 1% Triton X-100, 0.1% SDS, 0.1% sodium deoxycholate 1 mM Leupeptin, 1 mM Aprotinin, 20 mM PMSF). Lysates were subjected to PAGE electrophoresis and transferred to nitrocellulose. The membranes were blocked in 5% milk for 1 h and incubated overnight with the specified antibodies. Following incubation, membranes were washed three times in 0.1% Tween-20–TBS, and incubated for 1 hour with HRP anti-mouse or anti-rabbit IgG secondary antibodies (Sigma 1:2500). Membranes were then washed as above and proteins were detected using ECL (Promega, Fitchburg, WI, USA).

### Immunofluorescence (IF) staining

Cells were grown on glass coverslips in six-well plates. After washing with PBS, cells were fixed with three alternative methods as specified in respective figure legends, including ice-cold methanol for 10 min (fix with permeabilized cell membrane), 4% paraformaldehyde at room temperature for 15 min (fix with preserved cell membrane), and CSK extraction with 0.1% Triton X-100 CSK (10 mM PIPES pH 7.0, 100 mM NaCl, 300 mM sucrose, 3 mM MgCl_2_) buffer for 1 min followed by 4% paraformaldehyde (fix after extraction of soluble proteins). Following fixation, the coverslips were blocked in 0.3% Triton, 2% bovine serum albumin for 1 hour (immunofluorescent block buffer), immunostained overnight with the indicated antibodies in blocking buffer, washed 3-times in PBS + 0.1% Triton X-100 for 5 min, and then incubated with 1:1000 dilution of FITC or TRITC conjugated anti-mouse or anti-rabbit secondary antibodies (1:1000, Sigma) for 1 h in blocking buffer. After washing, the glass slips were mounted onto slides with Vectashield mounting media containing 4',6-diamidino-2-phenylindole (DAPI).

To stain cells after RNase and DNase treatments, cells on coverslips were rinsed once with PBS, permeabilized by incubating for 5 min at RT with 0.1% Triton X-100 CSK buffer, treated for 20 min at RT with either PBS (mock treatment) or RNase A (ThermoFisher, EN0531) or DNase I (ThermoFisher, EN0525), rinsed with PBS, and fixed with 4% paraformaldehyde for 15min at RT. The cells were blocked and cultured with antibody described like above. RNA and DNA were stained with 500 nM of Pyronin Y for 30 seconds or 1 μg/ml of Hoechst 33342 for 2 min at RT respectively.

### Polysome fractionation analysis

During the study, we used two protocols for sucrose gradient analysis in the density ranges of 17–47% and 10–45%. For 17–47% sucrose gradient polysome analysis, we used the procedure described by Oh *et al.* (40). Briefly, MEFs, HT1080 or U2OS cells were cultured for 48 h with 10% serum. Two hours before the analysis, the cultures were changed to fresh warm medium, incubated with cycloheximide (100 μg/ml) for 20 min, harvested, and then lysed in hypotonic buffer (20 mM potassium acetate, 12 mM magnesium acetate, 20 mM Tris–HCl, pH 7.4) by Dounce homogenization (35 strokes). Cell debris and nuclei were removed by centrifugation for 5 times at 4 °C with 15 000 g for 5 min. After the optical density (OD) of the supernatant was measured at *A*_260_, 20 OD units of lysates were layered on top of a 10 ml 17–47% (wt/vol) sucrose gradient (10 mM sodium chloride, 12 mM magnesium chloride, 20 mM Tris–HCl, pH 7.4), and centrifuged for 4 h at 23 000 rpm in an AH-629 Sorvall rotor. The A260 was monitored and recorded using density gradient fractionator (Brandel, Gaithersburg, MD, USA). Fractions were concentrated to equal volume by Vivaspin concentrator (Sartorius, Elk Grove, IL). For 10–45% sucrose gradients, we used the procedure of Strezoska *et al.* (41). After collecting cells, the cells were lysed with buffer (20 mM Tris–HCl pH 7.2, 130 mM KCl, 10 mM MgCl_2_, 2.5 mM DTT, 0.5% NP-40, 0.5% sodium deoxycholate, 100 μg/ml cycloheximide, 0.2 mg/ml heparin, 200 U/ml RNasin) for 15 min on ice. The lysates were centrifuged at 12 000 g for 10 min, and the supernatants were layered on 10–45% sucrose density gradients in 60 mM KCl, 10 mM MgCl_2_, 10 mM Tris–HCl, pH 7.2. The gradients were centrifuged at 36 000 rpm for 3.5 h at 4°C in a Beckman SW41Ti rotor.

Pre-ribosome isolation and fractionation were performed on a 10–30% (wt/wt) sucrose gradient as detailed before (42). Briefly, the cells were scraped off the dish in PBS, resuspended in 1 ml LSB (10 mM HEPES–NaOH [pH 7.5], 2 mM MgCl_2_, 10 mM NaCl, 1 mM EGTA), incubated on ice for 10 min, and centrifuged at 1200 × g for 5 min at 4 °C. The pellet was washed with 1 ml LSB containing protein inhibitors and lysed by adding NP40 (to a final concentration of 0.3%) and sodium deoxycholate (to a final concentration of 0.2%) for 30 s, centrifuged at 2800 × g for 5 min, and washed with 1 ml LSB again. The nuclear pellet was lysed with 300 μl HSB (10 mM Tris–HCl [pH 7.2], 0.5 M NaCl, 50 mM MgCl_2_, 0.1 mM CaCl_2_) containing 20 U Superasin RNase inhibitor and 150 U DNase I (Worthington, DPRF grade) for 10 min at room temperature. After centrifugation at 12 000 × g for 10 min, the pellet was briefly rinsed with 100 μl of cold NEB (10 mM Tris–HCl [pH 7.2], 10 mM NaCl, 10 mM EDTA) and centrifuged for 1 min. The pellet was extracted with 500 μl NEB containing 10 mM DTT and 0.2% Genapol C-100 at room temperature for 10 min, and then centrifuged at 12 000 × g for 10 min. The supernatant containing pre-ribosomes was analyzed on 10–30% (wt/wt) sucrose gradients made in NEB with 1 mM DTT and 0.01% Genapol C-100.

### Cellular fractionation

The cultured cells were collected by trypsin digestion and washed with cold PBS, incubated 10 min on ice with permeabilization buffer (10 mM HEPES pH 7.4, 10 mM KCl, 0.05% NP-40). Samples were centrifuged at 5000 × g for 5 min at 4°C. Supernatants were collected and used as the cytoplasmic fractions (CP), and pellet were washed once with permeabilization buffer, and centrifuged at 5000 × g for 5 min at 4°C. Supernatants were discarded and pellets were dispersed with CSK buffer on ice for 10 min and centrifuged at 5000 × g for 5 min at 4°C. The resultant supernatants were collected and used as the nuclear fractions (NP), and the remaining pellets were washed with CSK buffer and centrifuged at 5000 × g for 5 min at 4°C. Supernatants were discarded, and the pellets were suspended with buffer S1 (0.5 M sucrose, 3 mM MgCl_2_ with protease inhibitors), sonicated on ice at 50% power, 10 s on and 10 s off for five cycles, under-layered with buffer S2 (1 M sucrose, 3 mM MgCl_2_ with protease inhibitors), and centrifuged at 1800 × g for 5 min at 4°C. The resultant pellets were saved as the nucleoli fraction (No), and the supernatants contain the non-nucleolar nuclear fraction as well as some insoluble cytoplasm components. Alternatively, the cell fractionation kit (Cell Signaling Technology #9038) was used to separate the nuclear fraction from the rest of the cell components using about 5 million of MEFs, and in parallel the same amount of MEFs was used to separate the nucleoli and remaining components by sucrose gradient centrifugation.

### RNA isolation and Northern blot analyses

Total cellular RNA was extracted using TRI Reagent (Molecular Research Center, Inc) according to the manufacturer's protocols. 1–2 μg of total RNA per lane was resolved on 1% agarose gels and analyzed by northern hybridizations with ^32^P-labeled oligonucleotide probes (Supplementary Table S1) followed by phosphor-imaging detection as described (43).

## RESULTS

### BCCIPβ localization in the nucleolus

**Figure 1.**
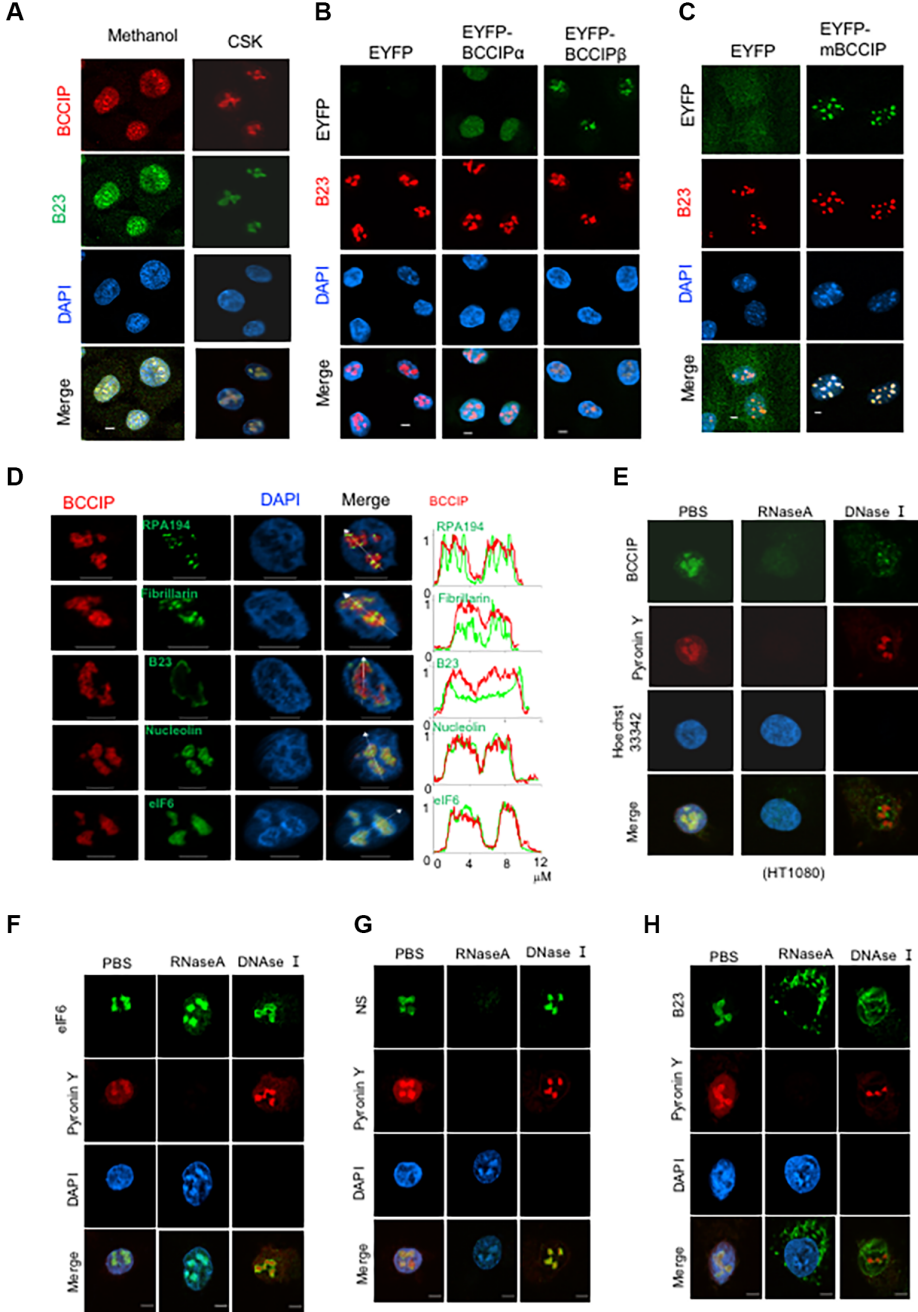
Nucleolus localization of BCCIP and its dependence on RNA. Other than specified, cells were permeabilized with CSK buffer for 1 min and then fixed with 4% of PFA prior to immunofluorescent staining. Scale bars = 5 μm. (**A**) Nucleolus localization of endogenous BCCIP in HT1080 cells. Anti-BCCIP (red) and B23 (green) staining were performed after direct fixation with methanol (left column), or after CSK-extraction followed by PFA fixation (right column) to visualize the location of endogenous BCCIP proteins. The verification of antibody specificity to endogenous BCCIP can be found in Supplementary Figure S1A. (**B**) BCCIPβ but not BCCIPα resides in the nucleolus. EYFP, EYFP-BCCIPμ, and EYFP-BCCIPμ were stably expressed in HT1080 cells and co-stained for B23 (red) after extraction with CSK buffer. The staining of total EYFP-BCCIPμ and EYFP-BCCIPβ protein can be found in Supplementary Figure S1B. (**C**) A fraction of mouse BCCIP resides in the nucleolus. EYFP or EYFP-mBCCIP were expressed in MEFs and stained with B23 antibody after extraction with CSK buffer. The staining of total EYFP-mBCCIP protein can be found in Supplementary Figure S1C. (**D**) Co-localization of BCCIP with different components of the nucleolus. The endogenous BCCIP protein (red) in HT1080 cells was co-stained with RPA194, Fibrillarin, B23, Nucleolin, and eIF6 respectively. After the confocal images were obtained, the relative signal intensities of BCCIP (red) and nucleolar protein markers (green) were measured along arbitrarily drawn arrowed lines, superimposed, and plotted in the right column. A co-localization of the two proteins would produce a largely overlapped signal distribution along the lines. The unit of abscissa in the right column are μm. (**E**–**H**) Dependence of the protein nucleolus localizations on RNA and DNA. Fresh HT1080 cells were treated with CSK buffer for 1 min, incubated with RNase A, DNase I or PBS for 20 min in room temperature, fixed with 4% PFA, and then stained with BCCIP, eIF6, nucleostemin (NS), and B23 antibodies before staining with Pyronin-Y (red) and Hoechst-33342 (blue) dyes.

Based on anti-BCCIP IF staining of cells, the majority of the total BCCIP protein was distributed throughout the nucleus (Figure 1A, left column). However, the Triton X-100 resistant BCCIP mainly resided in the nucleolus (Figure 1A, right column). The specificity of the BCCIP antibodies used in the IF staining was verified in Supplementary Figure S1A and also shown in a previous report (30). To further determine whether the human BCCIPα and/or BCCIPβ isoforms localize to the nucleolus, EYFP-BCCIPα and EYFP-BCCIPβ were independently expressed in human cells. As shown in Figure 1B, only the BCCIPβ isoform was detectable in the nucleolus after Triton X-100 extraction, while the majority of total EYFP-BCCIPα and EYFP-BCCIPβ was localized in the nucleus (Supplementary Figure S1B). In addition, the mouse BCCIP, which is conserved with the human BCCIPβ, also localized to the nucleolus (Figure 1C, Supplementary Figure S1C).

The mammalian nucleolus is composed of three major sub-compartments (44), including the fibrillar center (FC), the dense fibrillar component (DFC) and the granular component (GC). To determine whether BCCIP preferentially localizes to a specific sub-compartment, BCCIP was co-stained with RPA194 (a FC marker), Fibrillarin (a DFC marker), B23 (a GC marker), and Nucleolin (FC and DFC marker). As shown in Figure 1D, BCCIP had complete co-localization with Nucleolin, while it had only partial co-localizations with RPA194, Fibrillarin, and B23. This suggests that BCCIP is associated with the FC and DFC, where rRNA transcription and processing and early pre-ribosome assembly occur (44). Interestingly, we also found that BCCIP largely co-localized with eIF6 in the nucleolus (Figure 1D, bottom row).

Since the nucleolus is rich in ribosomal DNA (rDNA) and rRNA, we wanted to know whether BCCIP’s nucleolar retention is dependent on rDNA or rRNA. As indicated by Pyronin Y staining (Figure 1E), treatment with RNase A completely abrogated both RNA and BCCIP signals in the nucleolus, indicating that BCCIP retention in the nucleolus is dependent on nucleolar RNA. The treatment with DNase I also dramatically altered the nuclear BCCIP staining pattern (Figure 1E). Notably, it did not affect eIF6 (Figure 1F), suggesting that once eIF6 is recruited to the nucleolus, its retention in the nucleolus is no longer dependent on RNA or DNA. RNase treatment had the same effect on nucleostemin (NS) localization as on BCCIP but DNase treatment had little effect (Figure 1G). Consistent with a previous report (45), both the RNase and DNase treatments disrupted the nucleolar distributions of B23 (Figure 1H).

### BCCIP is required for biogenesis of 60S ribosomal subunits in mammalian cells

**Figure 2.**
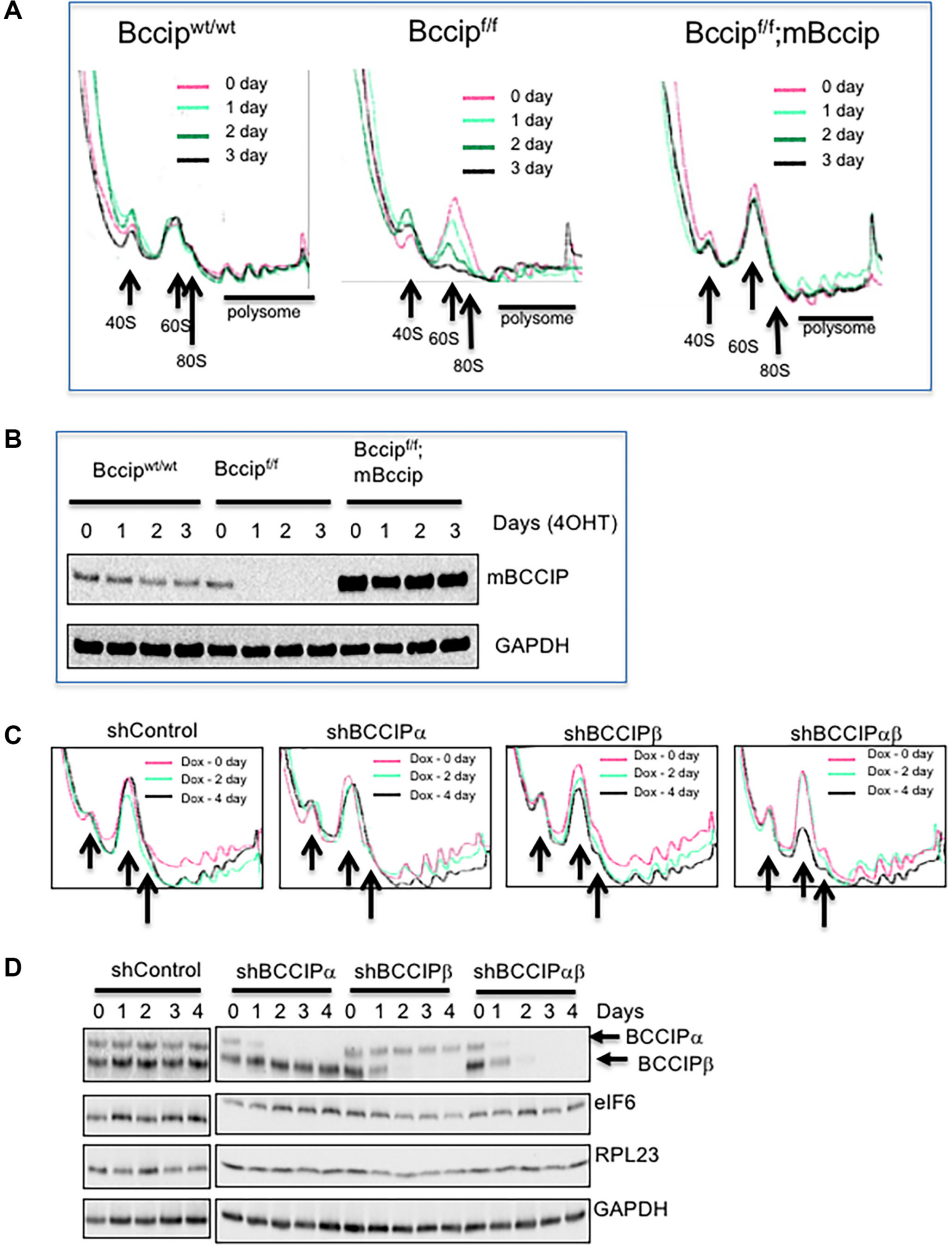
Defective 60S ribosomal subunit biogenesis in *Bccip* deficient cells. (**A**) Polysome profiles in MEFs after induced *Bccip* deletion. *Bccip^wt/wt^;Rosa-CreERT2 (Bccip^wt/wt^), Bccip^f/f^;Rosa-CreERT2 (Bccip^f/f^)*, and *Bccip^f/f^;Rosa-CreERT2;mBCCIP (Bccip^f/f^;mBCCIP)* cells were treated by 0.5 μM 4-hydroxy-tamoxifen (4OHT) for the indicated length of time, washed, and then collected for total cell extracts for sedimentation assay. The *Bccip^f/f^;Rosa-CreERT2;mBCCIP cells* were made by infecting the *Bccip^f/f^;Rosa-CreERT2* cells with retroviruses carrying pLXSP-mBccip. The ribosome traces at different times after 4OHT exposure are marked with distinct colors. The location of 40S, 60S, and 80S (monosomes) are indicated by arrows. (**B**) Verification of induced depletion of mouse BCCIP proteins in Rosa-CreERT2 positive cells. Cells were exposed to 4OHT with the indicated length of time and then collected for western blots. 1) Bccip^wt/wt^: MEFs with wild type alleles; 2) Bccip^f/f^: MEF with homozygous floxed Bccip alleles; 3) Bccip^f/f^/mBccip: the above cells with exogenous expression of mBccip from a plasmid. (C and D) Effect of induced BCCIP knockdown in HT1080 cells on the ribosome levels. HT1080 cells with doxycycline (Dox)-inducible BCCIP down-regulation were treated with 200 ng/ml of Dox for the indicated lengths of time, and then the whole-cell extracts were used for polysome profiling (**C**) and Western blots to detect the indicated proteins (**D**). The location of 40S, 60S and 80S (monosomes) are indicated by arrows in panel C.

Since BCCIP physically localizes in the nucleolus, we performed polysome profiling in *Bccip* null cells to determine if its depletion affects cellular ribosome levels. A set of immortalized MEFs (see Supplementary Figure S2) were used for polysome profiling. There was little change in polysome profiles in *Bccip^wt/wt^;Rosa-CreERT2* cells after 4OHT treatment (left panel, Figure 2A). However, there was a gradual reduction of the 60S ribosome fraction after 4OHT treatment of the *Bccip^f/f^;Rosa-CreERT2* cells (middle panel, Figure 2A). When exogenous mouse *Bccip* was re-expressed in the *Bccip^f/f^;Rosa-CreERT2* cells, the 60S deficiency was rescued (right panel, Figure 2A). The effectiveness of 4OHT-induced BCCIP protein depletion was verified by western blot with the same cell extracts (Figure 2B). Treatment with Tamoxifen of the *Bccip^f/f^;Rosa-CreERT2* MEFs yielded similar results as the 4OHT treatment (Supplementary Figure S3). Using a different sedimentation gradient analysis protocol to resolve individual ribosomal species, we confirmed the selective decrease in free 60S ribosomal subunit levels, as compared to free 40S (Supplementary Figure S4). This was accompanied by a decrease in 80S monosomes and polysomes (Supplementary Figure S4). To further determine whether BCCIP deletion affects 60S biogenesis *in vivo*, the liver tissues of the *Bccip^f/f^;CreERT2^+^* mice were analyzed. As represented in Supplementary Figure S5, *Bccip* deletion effectively reduced the 60S subunits. Thus, these findings suggest that BCCIP is required for 60S but not 40S ribosome biogenesis in mice.

Unlike in mice, human cells express *BCCIPα* and *BCCIPβ* isoforms due to alternative splicing of the *BCCIP* RNA (46). To determine whether human BCCIP is also involved in 60S biogenesis, we used the previously established doxycycline (Dox)-inducible isoform specific *BCCIP* knockdown HT1080 cells (30). As represented in Figure 2C, knockdown of *BCCIPα* had little effect on the relative abundance of monomeric 60S subunits, and knockdown of *BCCIPβ* alone had a slight effect on 60S subunits. However, co-knockdown of both *BCCIPα* and *BCCIPβ* significantly reduced the levels of 60S ribosomal subunits in HT1080 cells. The effectiveness of Dox-induced *BCCIP* depletion was verified by western blots using the extracts of the same cells (Figure 2D). Furthermore, the effect of *BCCIP* knockdown on 60S ribosome biogenesis was detected both in HT1080 and U2OS cells with a different sucrose gradient scheme (10–45%) (Supplementary Figure S6). These data confirmed that human *BCCIP*, especially the *BCCIPβ* isoform, is required for 60S biogenesis.

Previously, it was reported that transient *BCCIP* knockdown in HeLa cells had no effect on ribosome biogenesis (37). To more closely examine how expression levels of BCCIP could affect ribosome biogenesis, we compared ribosome profiles among MEFs with homozygous and heterozygous *Bccip* deletions, and with inducible *Bccip* knockdown. As shown in Supplementary Figure S7A, the heterozygous Bccip^+/−^ MEFs had no detectable defect in 60S biogenesis, the shBccip MEFs had a drastic reduction of 60S but a small amount of 60S could still be generated, while the homozygous *Bccip* deletion caused an almost complete depletion of 60S. In agreement, both *Bccip^+/^^−^* and s*hBccip* MEFs are growth competent, and only the *Bccip^−^^/−^* MEFs were fully growth arrested (Supplementary Figure S7C). Together, these data (Figure 2, Supplementary Figures S2–S7) reveal that optimal levels of BCCIP are required for sufficient 60S biogenesis and to sustain cell viability.

### Interaction of BCCIP with eIF6

**Figure 3.**
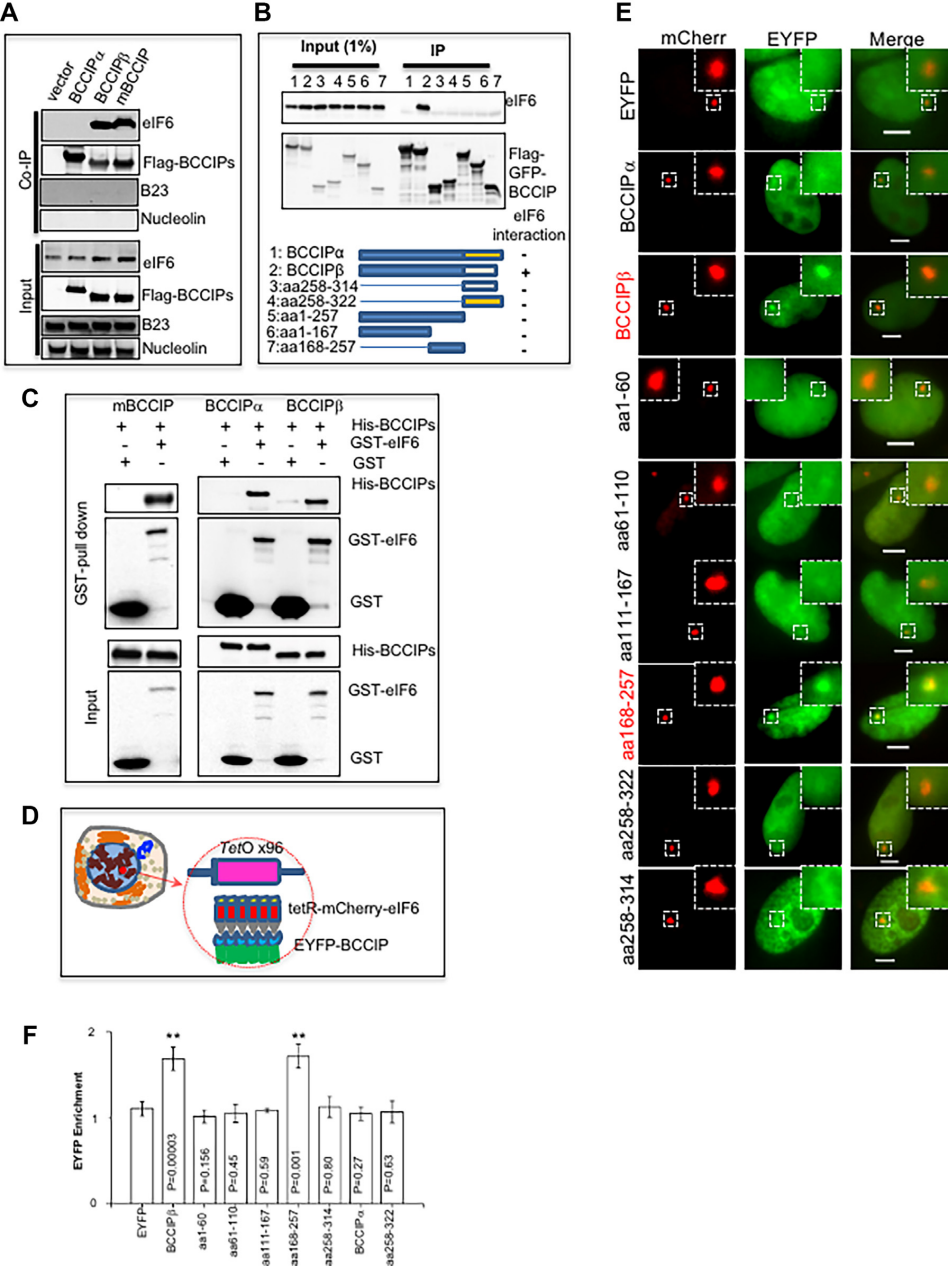
BCCIP interaction with eIF6. (A, B) Co-precipitation of eIF6 with BCCIPβ. Flag-tagged exogenous proteins were expressed in HEK293 cells after transient transfections of plasmids expressing the indicated BCCIP proteins (**A**) or truncated BCCIPβ (**B**). After 56 hours, cells were lysed for a co-precipitation assay with M2 anti-Flag beads. The co-precipitated eIF6 was detected by western blots. Bottom panel of (B) illustrates the regions of truncated BCCIP. (**C**) Interaction of purified recombinant BCCIP and eIF6. His-tagged mBCCIP, BCCIPα, and BCCIPβ proteins were expressed in E. coli, incubated with recombinant GST or GST-eIF6 proteins, and then precipitated with glutathione beads. The co-precipitated His-BCCIP proteins were detected by western blots. (**D**) A co-localization approach to visualize protein interaction in live cells. The U2OS/TRE cells contain an integrated copy of TetO repeat sequences in the X-chromosome. A protein fused with TetR-NLS-mCherry (such as eIF6) can be recruited to specific sites by TetO sequences and visualized as a single red dot in the nucleus. When an EYFP-fused second protein (such as BCCIP) binds with the first protein, the EYFP green signal will be enriched and colocalized with the red nuclear dot. (E, F) Mapping the BCCIP domain that binds with eIF6. eIF6 was fused with TetR-NLS-mCherry and expressed in U2OS/TRE. Full-length and various versions of truncated BCCIP were fused with NLS-EYFP and co-expressed. (**E**) Shown are representative images taken with a fluorescence microscope. Inserts are the zoomed areas surrounding the TetO array. Scale bars represent 5 μm. The relative co-localization intensity of BCCIP fragments with eIF6 measured in multiple cells is shown in (**F**). The *P*-values (as compared to EYFP control) are shown inside the bars of panel F. **P< 0.01.

Using immuno-precipitation, we found that both human BCCIPβ and mouse BCCIP can pull down eIF6, but human BCCIPα cannot (Figure 3A). Interestingly, although BCCIP colocalized with Nucleolin in the nucleolus (Figure 1D), BCCIP failed to immuno-precipitate Nucleolin or B23 (Figure 3A). Because BCCIPα and BCCIPβ have an identical N-terminal 257 amino acid sequence, and mouse BCCIP is homologous to human BCCIPβ, it is possible that the eIF6-interacting domain could reside in the C-terminal region of human BCCIPβ and mouse BCCIP. However, our truncation analysis suggested that the C-terminus of BCCIPβ was insufficient to co-precipitate eIF6 (Figure 3B). Recombinant GST-eIF6 pulled down mouse BCCIP, BCCIPβ (Figure 3C). It is interesting to note that the human BCCIPα isoform cannot be co-precipitated with endogenous eIF6 but can be co-precipitated with the recombinant GST-eIF6 (Figure 3C). Thus, it is likely that the C-terminal domain of BCCIPβ does not contain the eIF6 binding site, but rather is a regulatory domain *in vivo*.

Next, an intracellular method was adapted to investigate the interaction between eIF6 and BCCIP in live cells. A U2OS cell line, designated U2OS/TRE, had been generated with a single locus of 90 kb-long genomic DNA that contains multiple copies of a 96× tandem repeat of the tetracycline responsive element (Figure 3D) (47). A TetR-fused mCherry-eIF6 protein can be recruited to this locus and form a visible red dot in the cell (Figure 3E, left column). Then, different versions of truncated BCCIP were fused with EYFP and co-expressed in the cells. A co-localization of the EYFP with the mCherry in the same nuclear region would indicate an interaction between the eIF6 and the BCCIP. As shown in Figure 3E and F, the full-length BCCIPβ, but not BCCIPα, co-localized with eIF6, confirming the interaction between eIF6 and BCCIPβ in live cells. Likely due to an enhanced detection sensitivity with live cells (Figure 3E), as compared with the co-precipitation using cell extracts, the co-localization approach in Figure 3E was able to show that the amino acid region 168–257 of BCCIP was sufficient to colocalize with eIF6. Combining these results with the analysis of purified proteins (Figure 3C), our findings suggest that the physical interaction with eIF6 is through a common region between the BCCIPβ and BCCIPα, but the unique C-terminus region of BCCIPβ is required to enable the *in vivo* interaction, while the C-terminus unique region of BCCIPα is unable to perform this function *in vivo*.

### *BCCIP* is required for eIF6 localization in the nucleolus

**Figure 4.**
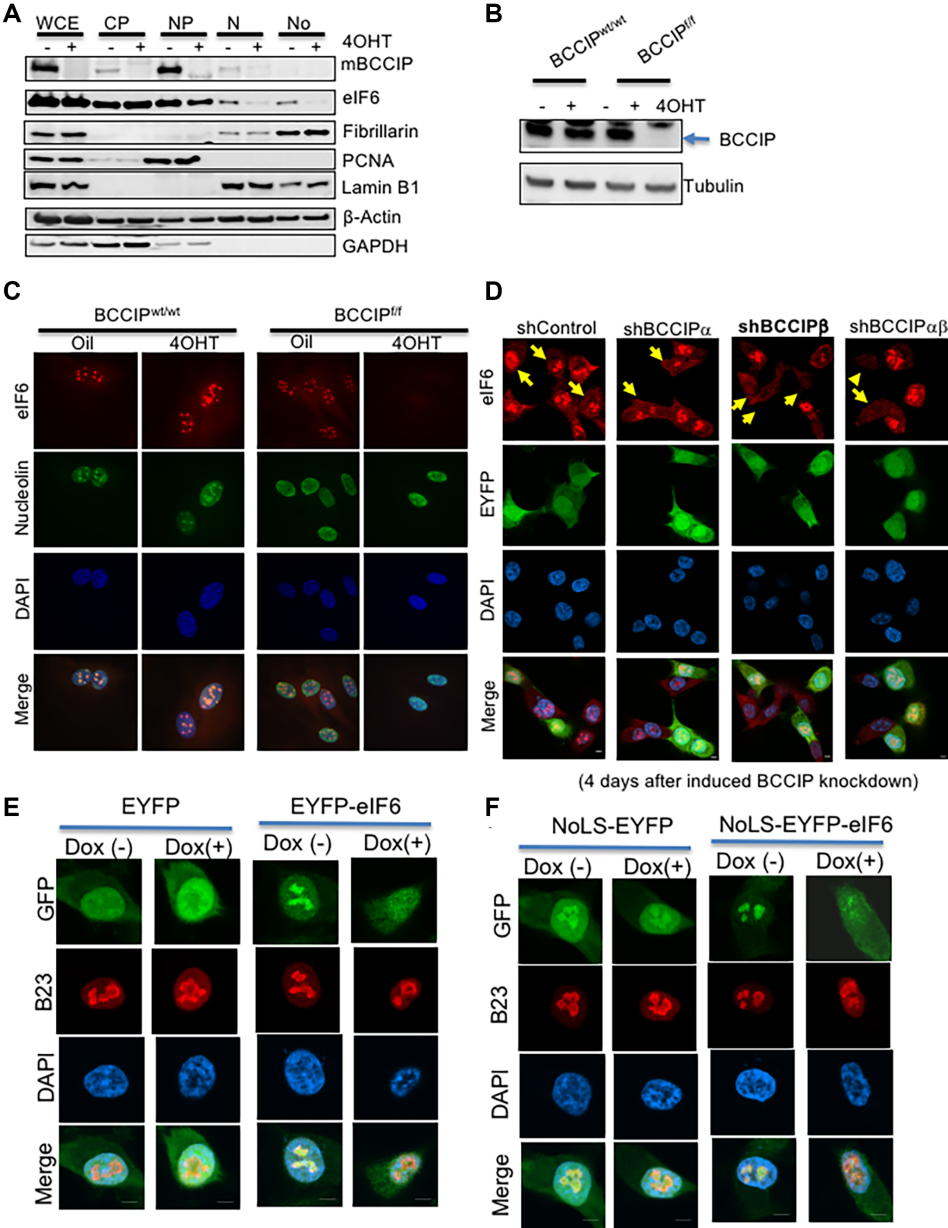
BCCIP is required for eIF6 localization in the nucleolus. (**A**) Cell fractionation analysis of protein distribution. *Bccip^f/f^;Rosa-CreERT2* cells were treated with 4OHT. Then, whole-cell lysates (WCE) were extracted from an aliquot of each cell population. The rest of the cells were fractionated to collect cytoplasm (CP), nuclear plasma (NP), insoluble nuclear fraction except the nucleolus (N), and nucleolus (No) fractions. These fractions were immuno-blotted to assess the relative level of indicated proteins. (**B, C**) *Bccip^f/f^;Rosa-CreERT2* cells were treated with 4OHT to induce the depletion of the BCCIP protein (**B**), and *BCCIP^wt/wt^;Rosa-CreERT2* was used as a control. These cells were stained with anti-eIF6 (red) and nucleolin (green) at day 4 (C). (**D**) Lack of nucleolar eIF6 in BCCIP-deficient human cells. The knock-downs of human BCCIPα and/or BCCIPβ isoforms were induced by Dox as shown in Figure 2D. These cells were mixed with EYFP-labeled wild type HT1080 cells, and stained for eIF6 (red) at different times after the doxycycline-treatment. Represented in panel D is the staining at day 4 after the Dox-treatment. Arrows indicate the non-green cells (with the indicated *BCCIP* knockdown, or the control shRNA). Staining at earlier time points after Dox-treatment can be found in Supplementary Figure S11. (**E**) Verification of the failed eIF6 recruitment to the nucleolus with EYFP-tagged protein. EYFP or EYFP-tagged eIF6 were expressed in HT1080 capable of Dox-induced BCCIP knock down (as shown Figure 2D). The cells were cultured with or without Dox for 3 days, and re-plated. The next day, the cells were fixed with 4% PFA, stained with B23 to locate the nucleolus, and observed to determine GFP-eIF6 localization. (**F**) Fusion of eIF6 with a nucleolus localizing sequence (NoLS) cannot force eIF6 recruitment to the nucleolus. NoLS-EYFP and NoLS-GFP-eIF6 were expressed HT1080 cells, the endogenous BCCIP in these cells was knocked-down by Dox treatment, and the localization of NoLS-tagged proteins was visualized as described in panel 4E.

Despite the interaction between mammalian BCCIP and eIF6, the total eIF6 level was not significantly affected by BCCIP depletion (Figure 2D) or over-expression (Supplementary Figure S8). Interestingly, upon BCCIP over-expression, we noticed an enhanced co-localization of eIF6 with human BCCIPβ and mouse BCCIP in the nucleus, but not with human BCCIPα (Supplementary Figure S9). This prompted us to investigate whether BCCIP regulates eIF6 intracellular distribution, which is considered critical to facilitate the 60S biogenesis. Using the inducible *Bccip* knockout in MEFs, we found that *Bccip* deletion significantly reduced eIF6 levels in the nuclear plasma, nucleolus, and insoluble nuclear fraction, without significantly affecting the level of eIF6 in total cell extract (Figure 4A). This observation was further supported by additional cellular fractionation assays (Supplementary Figure S10). Second, we observed a complete depletion of nucleolar eIF6 in the *Bccip* knockout cells (Figure 4B, C). In contrast, there was little change in the expression of nucleolin despite its complete co-localization with BCCIP (Figure 1D).

To verify this finding in human cells, we used the same panel of the human *BCCIP* isoform-specific, inducible knockdown HT1080 cells as described in Figure 2D, which showed that *BCCIP* was effectively knocked down at the second day after Dox exposure. Then, we stained eIF6 in these cells at different time points after the induction of BCCIP knockdown (Supplementary Figure S11). To stringently compare the relative eIF6 intensity between *BCCIP* knockdown and wild type cells on the same stained slides, EYFP-expressing *BCCIP* wild type cells were mixed with *BCCIP* knockdown cells, and the eIF6 signals were comparable between green (BCCIP wild type) and non-green (BCCIP knockdown) cells on the same slide. As shown in Figure 4D, at day 4 after Dox-induced knockdown, nucleolar eIF6 was dramatically reduced by knockdown of *BCCIPβ* and *BCCIPαβ* , however, little difference of eIF6 staining between the green (BCCIP wild type) and the non-green cells was observed following induction of shControl or shBCCIPα. This effect can be observed as early as day 3 (Supplementary Figure S11) when BCCIP knockdown became initially detectable (compare Supplementary Figure S11 with Figure 2D). The exogenous EYFP-tagged eIF6 displayed the same trend after down-regulation of endogenous BCCIP (Figure 4E). It has been shown that certain amino acid sequences, referred to as nucleolar localization sequences (NoLS), can help to retain some proteins in the nucleolus, but eIF6 lacks such a sequence. Thus, we tested whether fusion of a previously characterized NoLS (48) with eIF6 could force the retention of eIF6 in the nucleolus in the absence of BCCIP. As expected, the fusion of the NoLS successfully enriched NoLS-EYFP in the nucleolus while the EYFP itself was largely excluded from the nucleolus (compare Figure 4E with 4F, left panels). However, NoLS fusion with eIF6 failed to restore the nucleolar recruitment of eIF6 in BCCIP-depleted cells (Figure 4F). Furthermore, BCCIP deficiency did not influence the localization of RPL23, even if fused to the NoLS (Supplementary Figure S12), nor the overall stability of the eIF6 protein (Supplementary Figure S13). Together, these data firmly establish that BCCIP is required to retain eIF6 in the nucleolus.

### An N-terminus acidic stretch of BCCIP regulates nucleolar localization of BCCIP and eIF6

**Figure 5.**
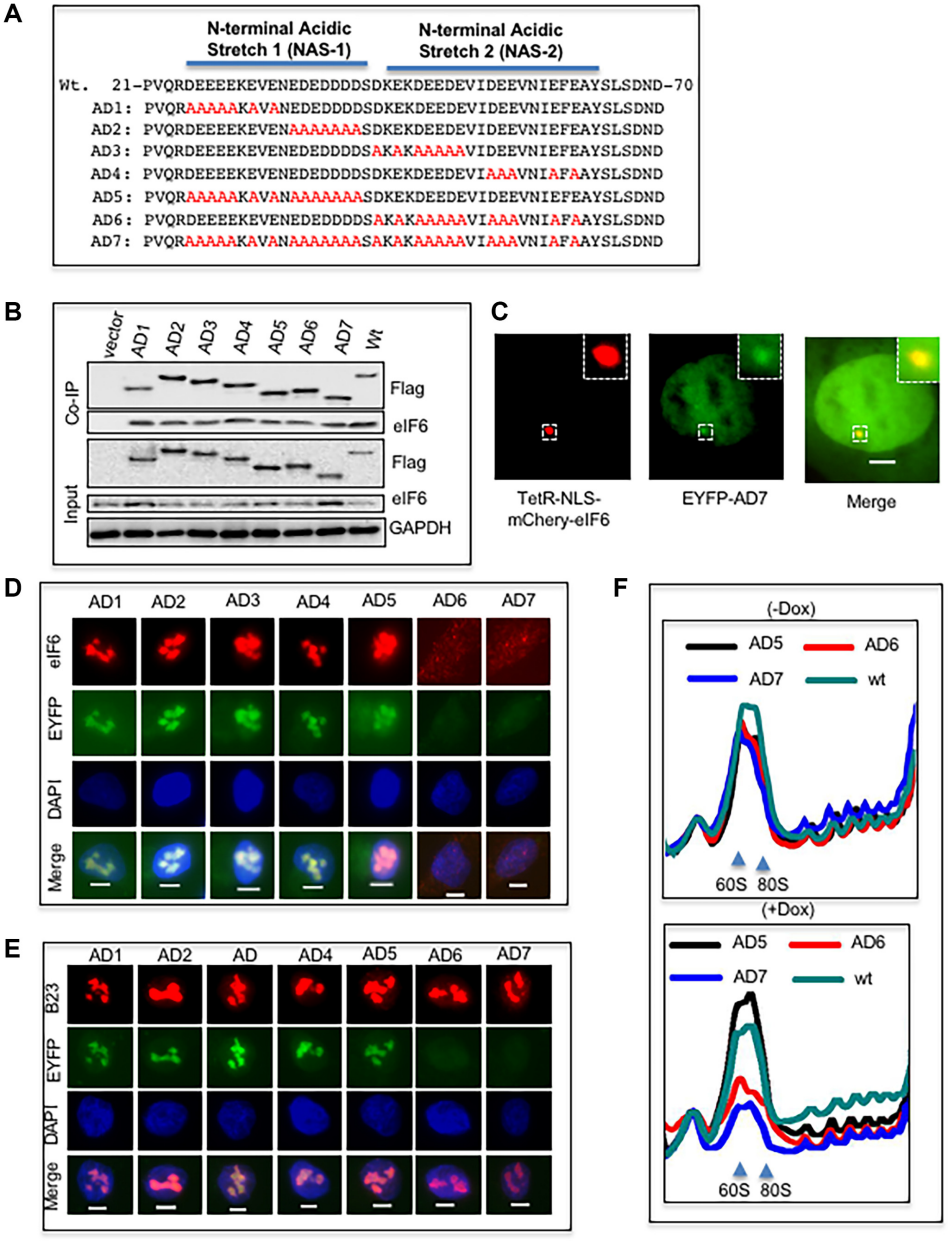
A stretch of acidic residues in the N-terminal domain of BCCIP is required for the nucleolus localization of BCCIP and eIF6. (**A**) of mutated acidic residues in the N-terminal region of BCCIP. In seven engineered BCCIP mutants (AD1-AD7), multiple acidic residues (D or E) in the region of aa25–62 were converted to A (shown in red). These AD mutants were made from a cDNA frame that is resistant to the RNAi in HT180 cells. (B, C) Change of the acidic residues did not affect the binding between BCCIP and eIF6. Flag-tagged BCCIP AD-mutants were transiently expressed in HEK293 cells and precipitated with M2 anti-Flag beads. The co-precipitated endogenous eIF6 was detected by western blots (**B**). The AD7 mutant was tagged with EYFP and co-expressed with Tet-NLS-mCherry-eIF6 in the U2OS/TRE cells. The colocalization of these proteins is represented in (**C**). (D, E). The stretch of acidic residues in the region of aa43–62, but not in aa25–41, was required for the nucleolus localization of BCCIP, which is also required for eIF6 localization to the nucleolus. The shBCCIP-resistant AD mutants were expressed in HT1080 cells capable of Dox-inducible BCCIP knockdown (Figure 2D). After 3 days of Dox-treatment to deplete the endogenous BCCIP, the cells were pre-treated with CSK buffer, fixed with 4% PFA and then stained with eIF6 (**D**) or B23 (**E**). Representative images show the lack of nucleolus location of the AD6 and AD7 mutants, as well as eIF6, but not B23. (**F**) The AD6 and AD7 BCCIP mutants cannot rescue the 60S biogenesis defect. The same cells in (D were collected for polysome profiling. The wt and AD5 mutant (that was able to localize to nucleolus) were used positive controls for the rescue experiment. Top panel represents PBS treatment (–Dox) and bottom panel represents Dox treated cells.

A distinct feature of the BCCIP protein is the enrichment of acidic amino acids in the N-terminus, primarily between amino acids 25–60. A PSI-blast based secondary structure Prediction (PSIPRED) suggested that the N terminal-half of this stretch (aa25–41), termed N-terminal Acidic Stretch-1 (NAS-1), may form a helix structure, while the C-terminal half (aa43–62), termed N-terminal Acidic Stretch-2 (NAS-2) likely exists as a flexible unstructured loop. Accordingly, we mutated multiple acidic residues to alanine in several BCCIP mutants (Figure 5A), and tested whether the N-terminus acidic domain regulates BCCIP’s nucleolar localization and affects nucleolar recruitment of eIF6.

When transiently expressed (Figure 5B and C), the mutants did not alter the binding between eIF6 and BCCIP as determined by co-immunoprecipitation and the TetO dependent co-localization assay in living U2OS/TRE cells, although migration in SDS-PAGE was affected (Figure 5B). Expression of these mutants in cells with endogenous BCCIP depletion showed that mutations involving the NAS-2 region (mutants AD6 and AD7) abolished the localization of BCCIP in the nucleolus (Figure 5D), although mutants were still largely present in the nucleus (Supplementary Figure S14). The same mutations failed to enable the nucleolar recruitment of eIF6, but did not impair the localization of B23 (Figure 5D, E and Supplementary Figure S14). More importantly, mutations in the NAS-2 failed to support biogenesis of the 60S ribosome (Figure 5F). These data suggest that the recruitment of BCCIP to the nucleolus is dependent on the acidic domain, especially NAS-2, and this region is also critical for recruiting eIF6 to the nucleolus and for 60S biogenesis.

### BCCIP-dependent recruitment of eIF6 to the nucleolus is crucial for 60S biogenesis

**Figure 6.**
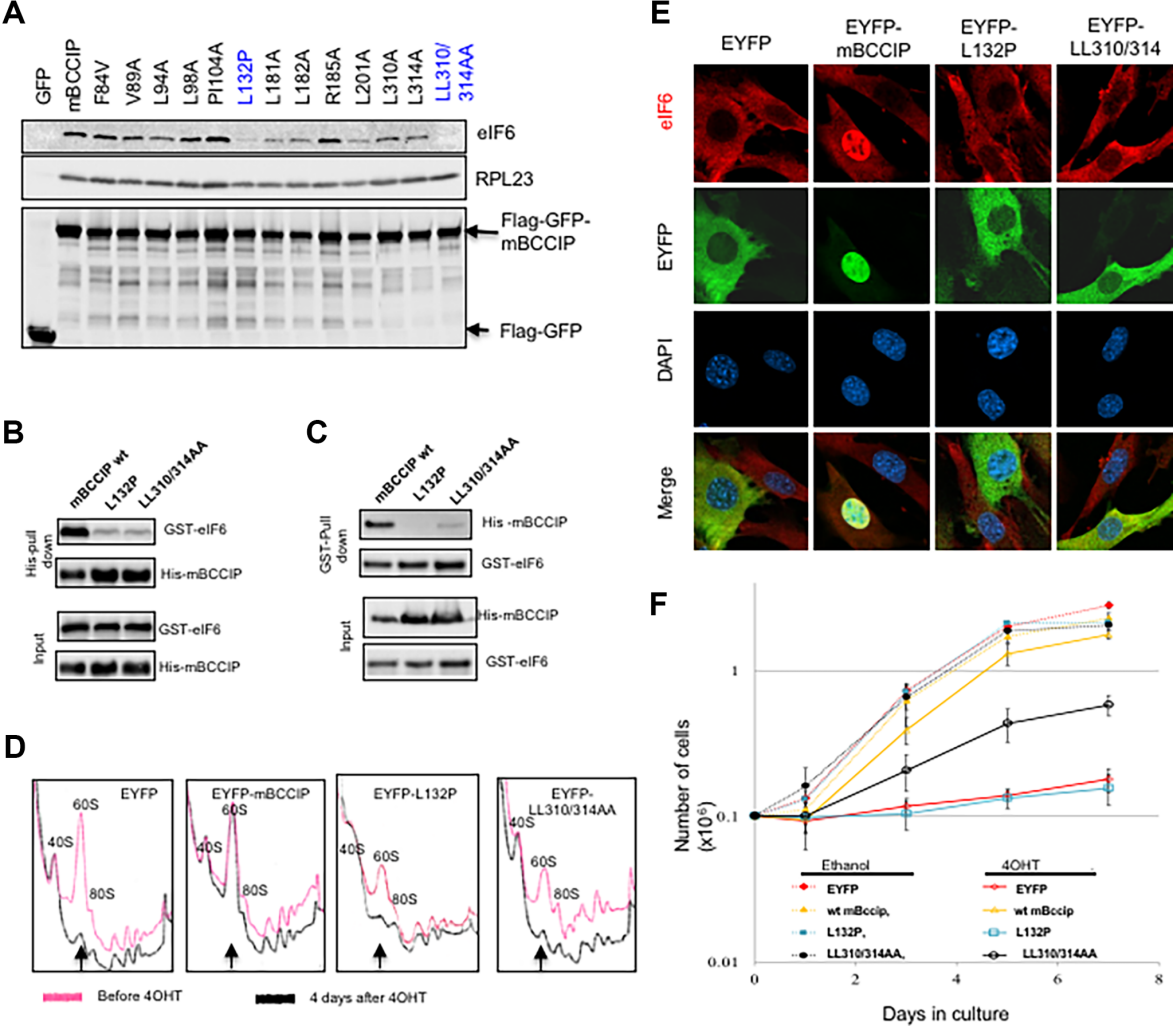
A weakened BCCIP-eIF6 interaction impairs nucleolar recruitment of eIF6 and 60S ribosome biogenesis. (**A**) Co-precipitation between eIF6 and BCCIP mutants. A panel of mouse BCCIP mutants as indicated were fused with Flag-GFP, and transiently expressed in NIH3T3 cells. The cell extracts were collected and precipitated with M2 anti-Flag beads. The co-precipitated endogenous eIF6 and RPL23 were detected by Western blot. GFP alone (left lane) was used as a negative control. (B, C) Impaired *in vitro* interaction between mutated recombinant mouse BCCIP with eIF6. Wild type, L132P mutant, and the LL310/314AA mutant were co-expressed in BL21 cells with GST-eIF6. After IPTG induced protein expression, the cell lysates were pulled down with His- (**B**) or glutathione beads (**C**). The co-precipitated proteins were detected by western blots. (D–F) Rescue of growth defect by BCCIP mutants in BCCIP-depleted MEFs. EYFP tagged wild type, mutant L132P, or LL310/314AA were stably expressed in *Bccip^f/f^;Rosa-CreERT2* cells respectively. EYFP alone was used as a control. Then the cells were treated with 4OHT to delete the endogenous *Bccip* gene, and used to determine polysome profiles (**D**), eIF6 localization (**E**) and the growth rates (**F**). In panel E, non-green *Bccip*-knockout cells were mixed with *Bccip* knockout cells expressing the EYFP-tagged exogenous *Bccip*, and stained for eIF6. Thus, all cells had depletion of endogenous BCCIP, but only the green cells had the expression of exogenous BCCIP proteins. As shown here, the expression of GFP-mBCCIP can enhance the localization of eIF6 to the nucleus (second column from left), while expression of EYFP, EYFP-L132P and EYFP-LL310/314 failed to enable the eIF6 recruitment in the nucleus after endogenous BCCIP depletion. In panel F. when comparing with EYFP control using *Student t*-test, the *P*-values among the 4OHT treated cells were: 0.0005 (wt. mBCCIP); 0.42 (mBCCIP-L132P) and 0.003 (mBCCIP-LL310/314AA). Among the ethanol treated cells, the P-values were: 0.56 (wt mBCCIP), *P* = 0.67 (mBCCIP-L132P) and *P* = 0.47 (mBCCIP-LL310/314AA).

Since eIF6 recruitment to the nucleolus is dependent on BCCIP (Figure 4) and mutations in the NAS-2 region were sufficient to abrogate eIF6 recruitment to the nucleolus and ribosome biogenesis (Figure 5), we tested the interaction between eIF6 and a panel of BCCIP mutants, including cancer relevant variants deposited in the TCGA database. Two mutants outside of the mapped eIF6 binding domain (aa167–258), human BCCIP-L308A&L312A, as well as mouse BCCIP-L132P (equivalent to human BCCIP-L130P) were of particular interest since they significantly reduced, but did not completely abolish, their interactions with endogenous eIF6 (Figure 6A), while their interactions with RPL23 were not affected. The reduced interaction between these mutants and eIF6 was confirmed with recombinant proteins (Figure 6B & C). Considering that aa168–257 of BCCIP were sufficient to bind to eIF6 in live cells (Figure 3E), and the BCCIPβ C-terminus is likely to be a regulatory element for BCCIPβ interaction with eIF6 but itself was unable to bind eIF6, we investigated whether these mutants can rescue the growth retardation in the *Bccip* knockout MEFs. We found that these mutants indeed do not rescue the 60S biogenesis defects and the recruitment of eIF6 to the nucleus (Figure 6D and E). After induced *Bccip* deletion, re-expression of Bccip-L132P failed to rescue growth arrest and Bccip-L310A&L314A only partially restored the growth capacity, while the wild type EYFP-mBccip fully rescued growth (Figure 6F). Taking these findings (Figures 5 and 6) together, it can be inferred that the regulation of the BCCIP-eIF6 interaction and BCCIP’s localization to the nucleolus are critical for eIF6 nucleolar recruitment, 60S ribosome biogenesis, and cellular growth. Of note is that these two mutants (Bccip-L132P, and Bccip-L310A&L314A) efficiently co-precipitate RPL23, yet do rescue the growth and 60S biogenesis defects (Figure 6).

### BCCIP is required for the generation of the 12S pre-rRNA

**Figure 7.**
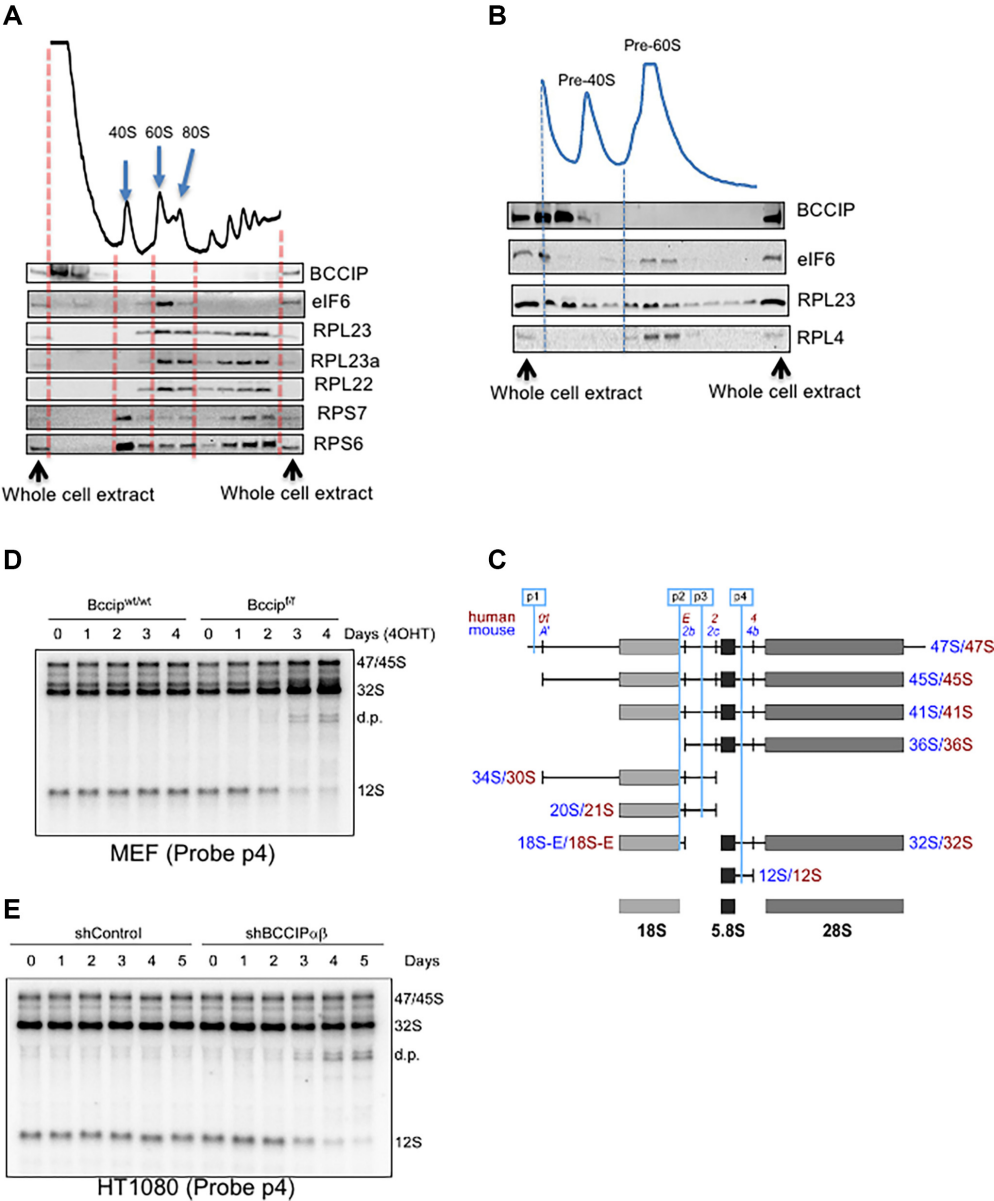
BCCIP regulates 60S subunit biogenesis by modulating pre-rRNA processing. (A, B) Relative abundance of BCCIP and representative ribosomal proteins in mature (**A**) and pre-ribosome (**B**) fractions. MEFs were collected, and the cytoplasmic extracts (A) and pre-ribosome-containing nuclear extracts (B) were separated by sedimentation through sucrose gradients. Fractions were concentrated to an equal volume and analyzed by Western blotting with the antibodies for RPS3, RPL22, RPL23a, eIF6 and BCCIP. The lanes in the Western blots are aligned with the gradient fractions. The left and right lanes were loaded with whole-cell extract to assist the identification of the protein species and provide a relative abundance reference in the Western blot. (**C**) Illustration of the major pre-ribosomal RNA species (pre-rRNAs) in human and mouse cells. Positions of hybridization probes (p1-p4) relative to cleavage sites in pre-rRNA are shown at the top. See Supplementary Table S1 for the sequence of the probes. (D, E) Requirement of BCCIP for pre-rRNA maturation. *Bccip^wt/wt^;Rosa-CreERT2* MEFs and *Bccip^f/f^;Rosa-CreERT2* MEFs were treated with 4OHT for the indicated lengths of days (**D**). The HT1080 cells capable of Dox-induced BCCIP knockdown were treated with Dox for the indicated time (**E**). The cells were collected and their RNA was analyzed by Northern hybridization with probe p4. d.p., degradation products of the pre-rRNA. See Supplementary Figures S15 and S16 for hybridizations with additional probes.

We next asked whether the BCCIP protein was physically present in the 60S ribosome fractions. As expected, small subunit ribosomal proteins (RPS6 and RPS7) and large subunit ribosomal proteins (RPL22 and RPL23a) were enriched in the 40S and 60S fractions, respectively (Figure 7A). eIF6 co-sedimented with the 60S peak, but not with the 80S or polyribosome fractions, which is in agreement with previous observations that eIF6 is associated with pre-60S or mature 60S ribosomes. However, the BCCIP protein was not observed in any of these fractions, suggesting that BCCIP is unlikely to be a physical component of mature ribosomes. To address whether BCCIP is associated with pre-ribosome complexes, we fractionated nuclear extracts and probed for BCCIP and eIF6 in pre-ribosome fractions. As shown in Figure 7B, there was no detectable signal for BCCIP in the pre-60S fractions, while eIF6 was present. This is in a contrast to observations of the yeast Bcp1 protein, which was shown to co-reside with 60S subunits in a ribosome fractionation assay (35), but did not co-precipitate Tif6 (36).

Because pre-rRNA processing into mature rRNAs is an integral part of ribosome biogenesis (7), we next investigated whether BCCIP depletion affects pre-rRNA levels by Northern blot analysis in MEFs and human cell lines using probes specific for spacer regions of the mouse and human pre-rRNA transcripts (see Figure 7C and Supplementary Table S1 for the location and sequence of probes). We observed a pronounced decrease in the level of the 12S pre-rRNA, which is a precursor of 5.8S rRNA, upon induction of *Bccip* deletion by 4OHT in *Bccip^f/f^;CreERT2^+^* MEFs (Figure 7D,. In parallel to the 12S reduction, there was a gradual increase in the 32S pre-rRNA, the 12S precursor, and the appearance of a characteristic double band of degradation products, previously observed during abortive maturation of mammalian pre-60S ribosomal subunits (49). When BCCIP knockdown was induced in human HT1080 and U2OS cells by Dox, the same trend of 12S pre-rRNA reduction and degradation of the upstream intermediates was observed, but in contrast to MEFs, 32S pre-rRNA did not accumulate (Figure 7E). A more drastic reduction of 12S pre-rRNA and accumulation of 32S pre-rRNA was observed when HT1080 cells were kept in the knockdown condition for an extended time period (Supplementary Figure S15). Using additional probes (Figure 7C, Supplementary Table S1), we did not observe significant changes with other rRNA precursors (Supplementary Figure S16). Considering that the 12S pre-rRNA is a cleavage product of the 32S pre-rRNA (6,7), these data suggest that mammalian cells require BCCIP function during 60S maturation steps leading to the production of the 12S pre-rRNA from the 32S pre-rRNA. Recently, it was suggested that mammalian eIF6 may also be involved in the generation of 12S pre-rRNA (8).

## DISCUSSION

In this study, we demonstrated that both the human and mouse BCCIPs are critical for 60S ribosome biogenesis and the generation of 12S pre-rRNA. A fraction of mouse BCCIP and human BCCIPβ resides in the nucleolus. Both the interaction between BCCIP and eIF6, and BCCIP nucleolar localization are required for eIF6 recruitment to the nucleolus, 60S biogenesis, and cell proliferation. Prior to our report, the role of mammalian BCCIP in ribosome biogenesis had not been established, although it had been implicated by a siRNA screen using pre-rRNA processing as the endpoint (2). However, in that study, a direct measurement of ribosomes failed to confirm a role of *BCCIP* in ribosome biogenesis despite co-precipitation of BCCIP with RPL23 and eIF6 (37), possibly due to the use of a transient BCCIP knockdown approach. Our report offers comprehensive experimental evidence that mammalian BCCIP is indeed a critical factor for ribosome biogenesis.

Although yeast and mice only have *BCCIPβ* homologues, humans have evolved two alternative splicing isoforms, *BCCIPα* and *BCCIPβ*, with *BCCIPα* preferentially involved in microtubule dynamics (30). In this study, we have shown that it is the conserved *BCCIPβ* isoform in humans that is mostly responsible for its role in ribosome biogenesis. Although there is a general conservation of the ribosome core components and biogenesis mechanisms in eukaryotes, mammalian cells have evolved a much more complex system to regulate ribosome synthesis. More than 25% of the 286 previously catalogued mammalian pre-rRNA processing protein factors lack obvious homologues in yeasts (2). Interestingly, we identified several distinctive features between yeast *Bcp1* and mammalian *BCCIP*. First, using a temperature sensitive yeast mutant (Bcp1-Phe249Ser), it was suggested that yeast Bcp1 promotes 60S biogenesis by serving as a chaperone to stabilize Rpl23; yeast Bcp1 interacts with Rpl23 directly but not with Tif6 (36). In contrast, BCCIP co-immunoprecipitates both eIF6 and RPL23 (37) (also see Figure 6A), and BCCIP directly binds to eIF6 (Figure 3C). Second, Bcp1 can be readily shown to co-sediment with 60S in yeast (36), but we could not detect BCCIP in the mature or pre-ribosome 60S fractions, although we cannot rule out the possibility that BCCIP may weakly or transiently associate with pre-ribosomes to deliver eIF6 to pre-ribosomes. Third, a compromised interaction was found between the Bccip-L310A&L314A (human BCCIP-L308A&L312A) and endogenous eIF6, but not with RPL23 (Figure 6). Yet these mutants had compromised function in 60S biogenesis, suggesting that the RPL23 interaction with BCCIP alone may not account for the contribution of BCCIP to 60S biogenesis. Fourth, despite the clear dependence on BCCIP for the nucleolar recruitment of eIF6 (Figure 4, Supplementary Figure S11), RPL23 localization was not affected by BCCIP deficiency (Supplementary Figure S12).

In budding yeast, cryo-EM modeling and structural analyses have found that Tif6 binds to pre-ribosomes in close proximity to Rpl23 (18,19,50). Rpl23 is required for binding of Tif6 to pre-ribosomes (51,52), and Bcp1 chaperones Rpl23 but does not bind to Tif6 directly (36). Thus, it is likely that yeast Bcp1 may mediate Tif6 function by acting as a chaperone for Rpl23. However, in mammalian cells, BCCIP can directly bind eIF6. While we clearly showed that eIF6 was affected by BCCIP, and that BCCIP consistently co-precipitated RPL23 along with eIF6, we found little evidence that BCCIP can affect RPL23 so far (Supplementary Figures S8 and S12). Thus, the mechanistic interface between the assembly factor eIF6/Tif6, RPL23 and BCCIP/Bcp1 may be different in budding yeast and mammalian cells, suggesting that there may have been a functional switch during evolution between yeast Bcp1 and mammalian BCCIP. One possible scenario is that in mammalian cells, BCCIP may be able to bypass RPL23 and directly serve as a chaperone for eIF6. Obviously, this would need additional investigations.

We have shown that BCCIP depletion impaired the generation of 12S pre-rRNA (Figure 7, Supplementary Figure S15), which is similar to a recent report on eIF6 (8). eIF6 is also an essential gene, but cells do not need a large amount of eIF6 to support ribosome biogenesis (16). An ∼80% reduction of eIF6 via RNA interference was associated with nucleolar localization of the remaining eIF6, which was sufficient to support an almost normal level of rRNA synthesis (16). Similarly, a profound reduction of BCCIP was required to abrogate its function in 60S biogenesis, consequently inhibiting cell growth. Cells can tolerate haploinsufficiency of *Bccip* and continue to synthesize 60S at normal levels. Although an ∼90% *Bccip* down-regulation can clearly decrease the biogenesis of 60S in MEFs, the remaining level of 60S biogenesis was sufficient to sustain cell viability (Supplementary Figure S7). Coincidentally, nucleolar BCCIP constitutes only a small portion of the total cellular BCCIP protein (Figure 4A). This perhaps can explain why an siRNA-based transient *BCCIP* knockdown failed to disrupt 60S biogenesis in an earlier report (37). It also underscores the complex role of *BCCIP* in tumorigenesis, where a partial loss of *BCCIP* function generates genomic instability (23–25,27,28), and a subtle down-regulation of *BCCIP* was observed in multiple cancer types (33,46,53–56), whereas a persistent or complete loss of *BCCIP* may hinder tumor progression.

While this study establishes the interaction between mammalian BCCIP and eIF6, many features of this interaction appear to be dynamic and regulatable, and worthy of future study. First, humans have two isoforms, BCCIPα and BCCIPβ, that have a common region of aa1–258 and differ only in the C-termini. Co-precipitation experiments showed that only BCCIPβ could efficiently pull down eIF6 (Figure 3A and B), and co-localize with eIF6 in living cells (Figure 3E and F). This might suggest that the C-terminus of BCCIPβ is responsible for the interaction. However, purified recombinant BCCIPα and BCCIPβ can both pull down eIF6 *in vitro*, and the C-terminus of BCCIPβ was not able to bind to eIF6 by itself (Figure 3B). Thus, the aa1–258 likely contain a binding domain for eIF6, but this region alone was not sufficient to co-precipitate eIF6 from cell extracts (Figure 3B). Using a more sensitive method with living cells, we identified aa168–257 of BCCIP as the eIF6 interaction domain (Figure 3E and F). These observations raise the possibility that the C-terminus of BCCIPβ may have a regulatory role for BCCIP-eIF6 interactions *in vivo*. Indeed, when the leucines 310 and 314 were changed to alanines, the mutants displayed a reduced ability to co-precipitate and bind eIF6 (Figure 6A, B, C), and caused mis-localization of BCCIP (Figure 6E). Thus, we suggest the direct binding is likely mediated by aa168–257 but the entire sequence of BCCIPβ is needed for a permissive or optimal configuration of BCCIP to support 60S biogenesis.

Second, although the majority of the nuclear eIF6 is found in the nucleolus (Figure 4C–E, Supplementary Figure S11), it remains to be determined to what extent the lack of nucleolar eIF6 in BCCIP-depleted cells is due to a defect in eIF6 loading into pre-ribosomal complexes, loss of its binding to other nucleolar targets or to reduced nuclear import of eIF6. Notably, BCCIP knockdown did not completely abrogate levels of nuclear eIF6 signal, although eIF6 nucleolar enrichment was greatly diminished (Figure 4E, F). This indicates that the effect of BCCIP on the nucleolar recruitment of eIF6 is unlikely to be explained only by a lack of nuclear import of eIF6. Considering that the digestion of RNA removed BCCIP, but not eIF6, from the nucleolus (Figure 1E, F) and yet depletion of BCCIP led to a loss of nucleolar eIF6, it can be inferred that the initial nucleolar recruitment of eIF6, but not necessarily its nucleolar retention, is dependent on BCCIP.

Third, since the initial submission of this manuscript, a structural analysis of yeast Bcp1 has suggested that the C- and N-termini of Bcp1 are flexible but can bind to each other to form Bcp1 dimers (57). It is thus possible that the relative formation of dimers versus monomers *in vivo* could be another regulatory mechanism, which will need to be addressed in the future studies of BCCIP structure and interactions.

In summary, our studies have identified BCCIP as a critical mammalian factor for the 60S ribosome assembly. This function is mediated through its regulatory function on eIF6 and rRNA processing steps that produce the 12S pre-rRNA.

## Supplementary Material

gkaa1114_Supplemental_Files
