# Supplementary material for: BCCIP is required for nucleolar recruitment of eIF6 and 12S pre-rRNA production during 60S ribosome biogenesis
Source: Nucleic Acids Res. 2020 Nov 27;48(22):12817–32. doi: 10.1093/nar/gkaa1114 (PMC7736804; doi:10.1093/nar/gkaa1114)
Supplement: gkaa1114_Supplemental_Files [file gkaa1114_supplemental_files.zip › NAR-00644 Suppl Figs S1-S16.pdf]

Fig S1

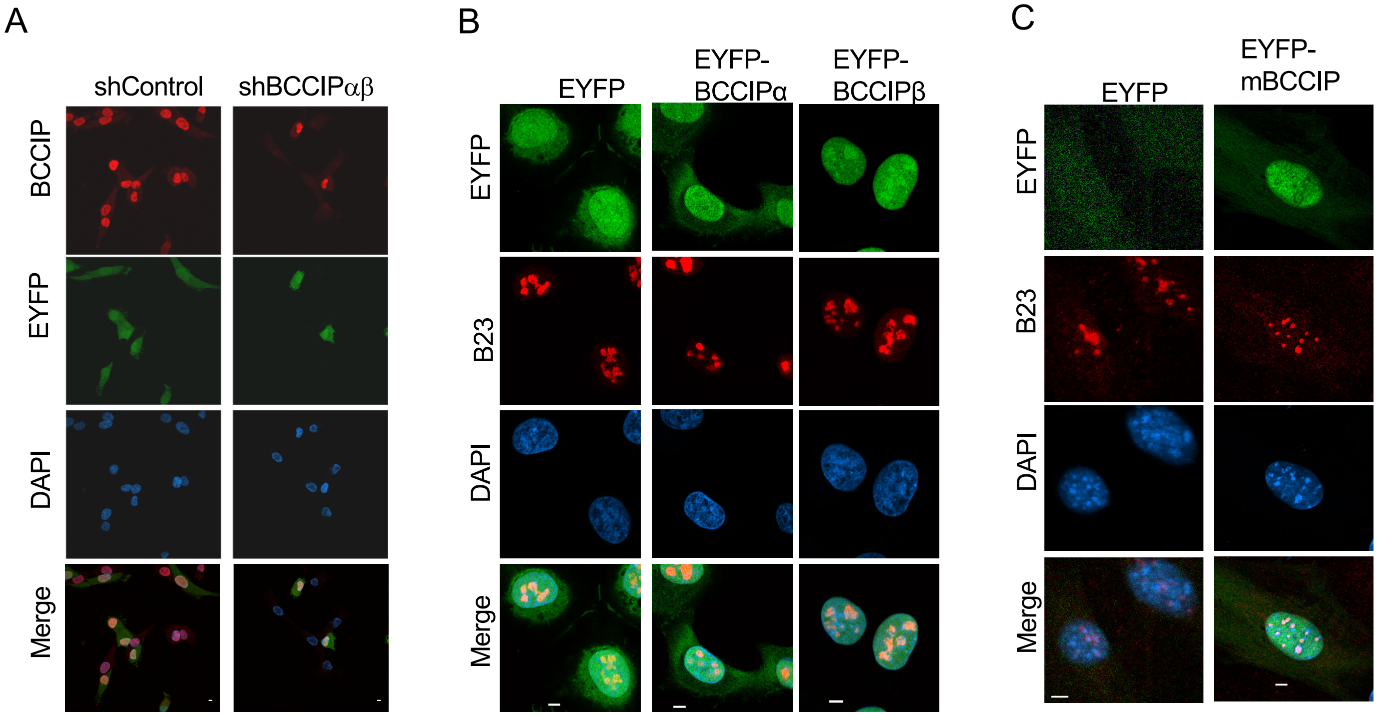

Fig S1. Antibody validation and the localization of total BCCIP protein in the cells.
(A): Validation of the antibody specificity for endogenous BCCIP in immuno-fluorescent staining. EYFP positive wild type HT1080 cells were mixed with non-fluorescent HT1080 cells that express control shRNA (shControl, left panels) or shRNA against both human isoforms of BCCIP (shBCCIPαβ, right panels). Then the mixed cells were stained with anti-BCCIP antibodies. As shown in the left panel, the EYFP positive and negative cells were equally positive of BCCIP staining (red). However in the right panel, only the EYFP positive cells were BCCIP positive and the non-fluorescent shBCCIP cells were negative of BCCIP staining. This confirmed that the antibody was highly specific for endogenous BCCIP protein.
(B, C): The nuclear localization of total human BCCIP. HT1080 cells (B) expressing EYFP, EYFP-BCCIPα, or EYFP-BCCIPβ, and MEFs (C) expressing EYFP or EYFP-mBCCIP were fixed with methanol, and then stained with B23 antibody (red). Scale bars represent 5 μm.

Fig S2

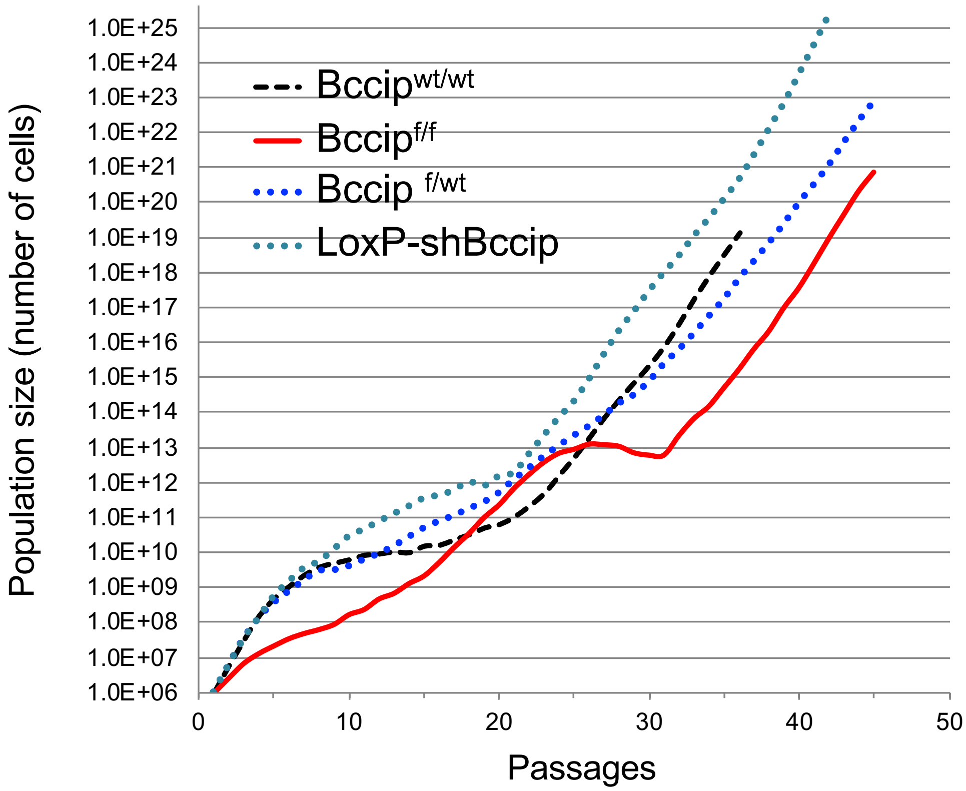

Fig S2. Population growth of immortalized mouse embryo fibroblasts (MEFs). Primary MEFs from the respective embryos were passed every three days at $1x10^6$ cells per 10cm dish. The population sizes were calculated based on the measured number of cells in the culture at each passing, the dilution factor at the time of re-plating, and the population size of the previous passage. Shown are population sizes of 4 MEF lines with the following genotypes: *1) $Bccip^{wt/wt}$;Rosa-CreERT2 ($Bccip^{wt/wt}$)*, 2) *$Bccip^{f/f}$;Rosa-CreERT2 ($Bccip^{f/f}$)*, 3) *$Bccip^{f/wt}$;Rosa-CreERT2 ($Bccip^{f/wt}$),* and 4) *flox-shBccip;Rosa-CreERT2 (LoxP-shBccip)* . These immortalized cells were growth competent for at least 40 passages and were used to perform ribosome sedimentation analyses through sucrose density gradients.

Fig S3

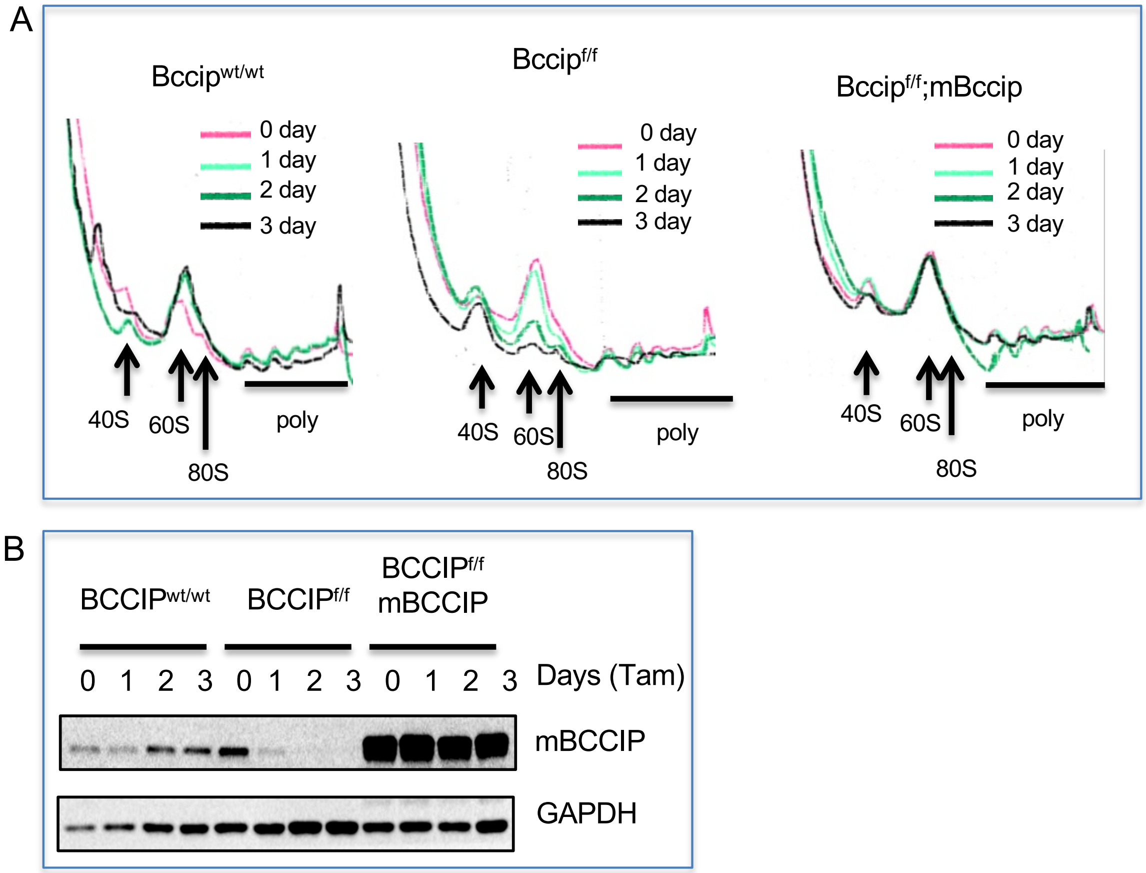

Fig S3. Verifications of impaired 60S biogenesis in conditional *Bccip* knockout MEFs by tamoxifen treatment. *Bccip$^{wt/wt}$;Rosa-CreERT2 (Bccip$^{wt/wt}$), Bccip$^{f/f}$;Rosa-CreERT2 (Bccip$^{f/f}$),* and *Bccip$^{f/f}$;Rosa-CreERT2;mBCCIP (Bccip$^{f/f}$;mBCCIP)* cells were treated with 1 μM Tamoxifen for indicated days, re-plated and cultured for 24 hours without Tamoxifen. The cells were treated with 100 μg/ml of cytoheximidine for 20 min and collected for ribosome profiles (A) and western blots (B). Tamoxifen did not influence 60S biogenesis in *Bccip* wild type (left panels of A), but 60S dramatically reduced in *Bccip$^{f/f}$* cells after Tamoxifen treatment (middle panels of A). Re-expression of exogenesis mBCCIP restores 60S biogenesis in the *Bccip$^{f/f}$* deletion cells (right panels of A).

Fig S4

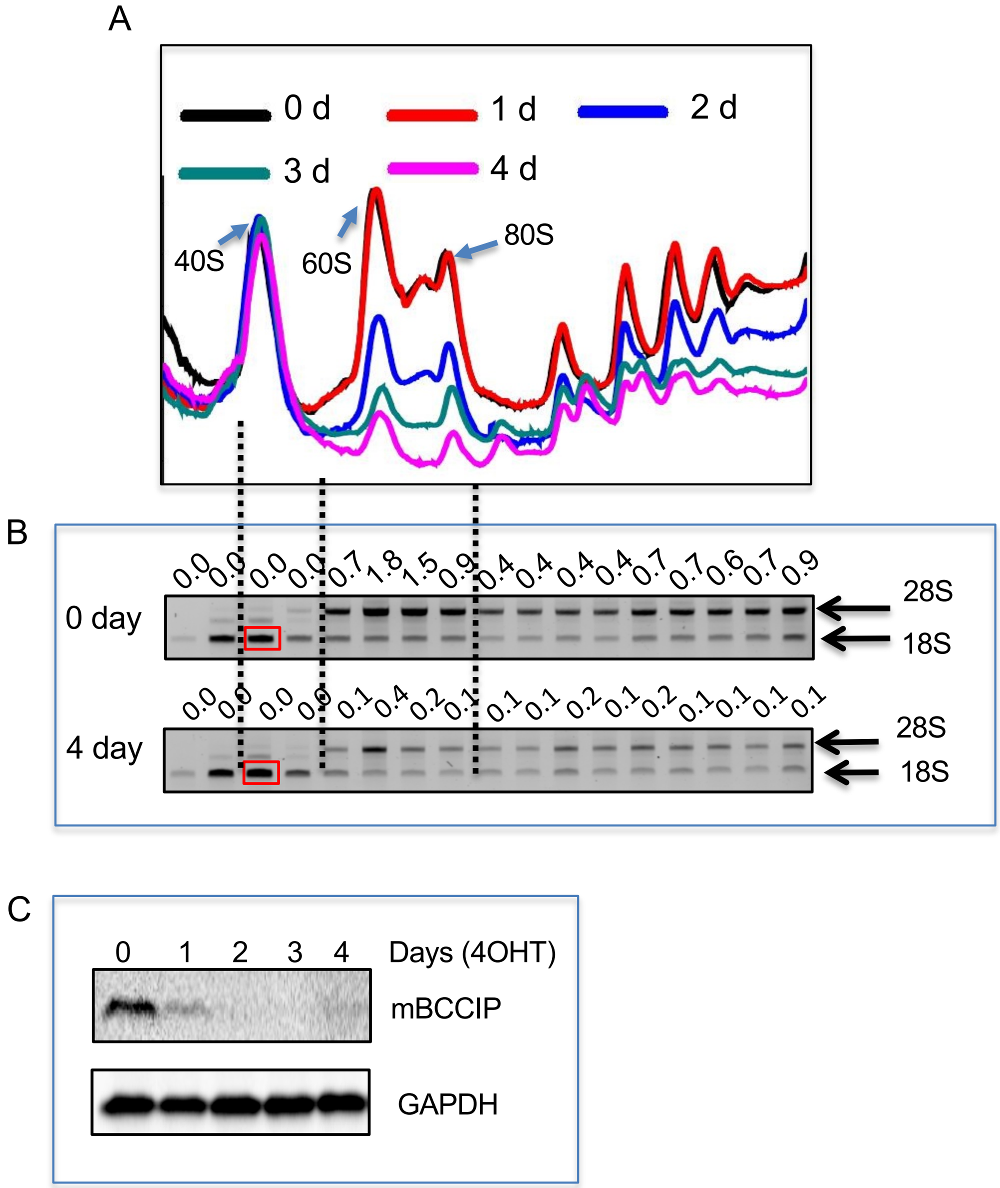

Fig S4. Verification of 60S ribosome loss by fractionation analysis of rRNA. *BCCIP*$^{f/f}$*;Rosa-CreERT2* cells were treated with 4OHT for indicated lengths of time and collected for ribosome profiling with a gradient range of 10%-45% (A). The RNA in each fraction of 4OHT treatment for 0 days and 4 days were extracted and resolved on agarose gels (B). The relative abundance of 28S in each fraction was normalized to the 18 sRNA of the 40S ribosome fraction (boxed in red) and labeled on top of each RNA gel. The efficiency of BCCIP depletion is shown in C.

Fig S5

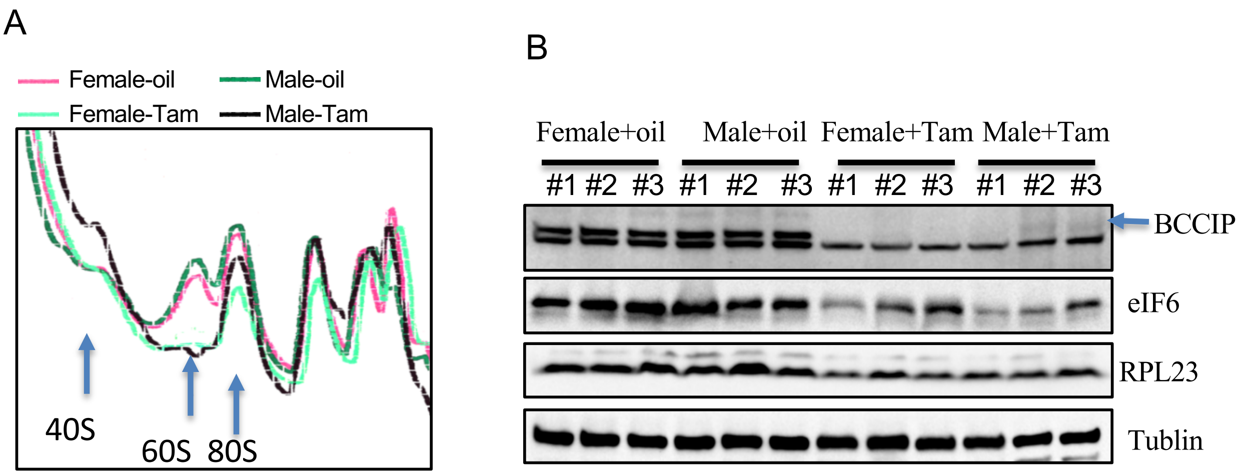

Fig S5. BCCIP regulates 60S ribosome biogenesis in liver. $Bccip^{f/f}$;Rosa26-$CreERT2^{+}$ mice were injected with oil or Tamoxifen. Three days after tamoxifen treatment of the mice, the liver tissues were collected for ribosome profiling, and western blots.

(A): Representative ribosome profiles of the liver extracts from tamoxifen (Bccip knockout) and oil treated littermate (control).

(B): Verification of mBCCIP depletion in mouse livers by western blotting. Shown are representative western blots of liver tissue extract from three pairs of littermate females and males.

Fig S6

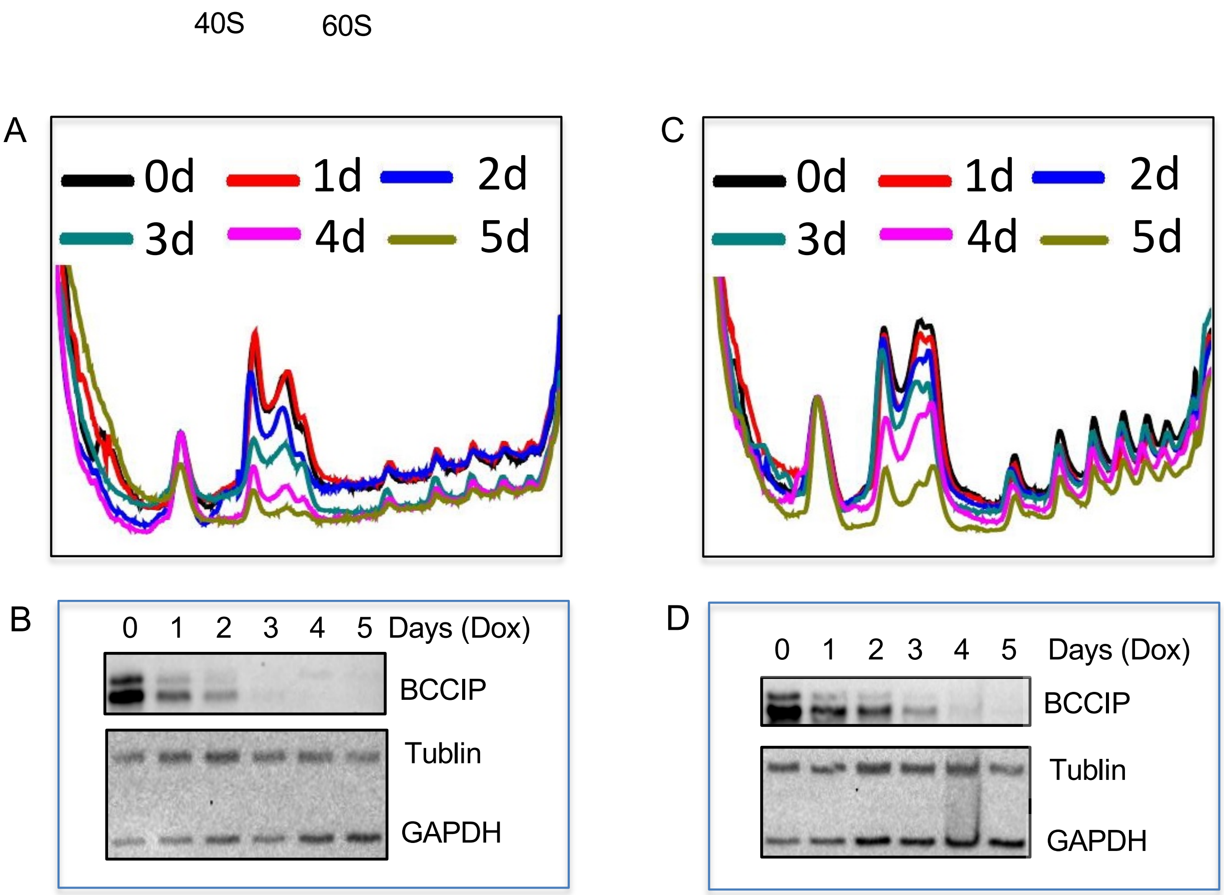

Fig S6. Verification of 60S ribosome loss in HT1080 and U2OS cells with inducible BCCIP knockdown. The cells with BCCIP inducible knock-down were treated with Dox for indicated lengths of time both in HT1080 (A, B) and U2OS (C, D) and collected for ribosome profiling with 10-45% gradient (A, C) and western blots (B, D).

Fig. S7

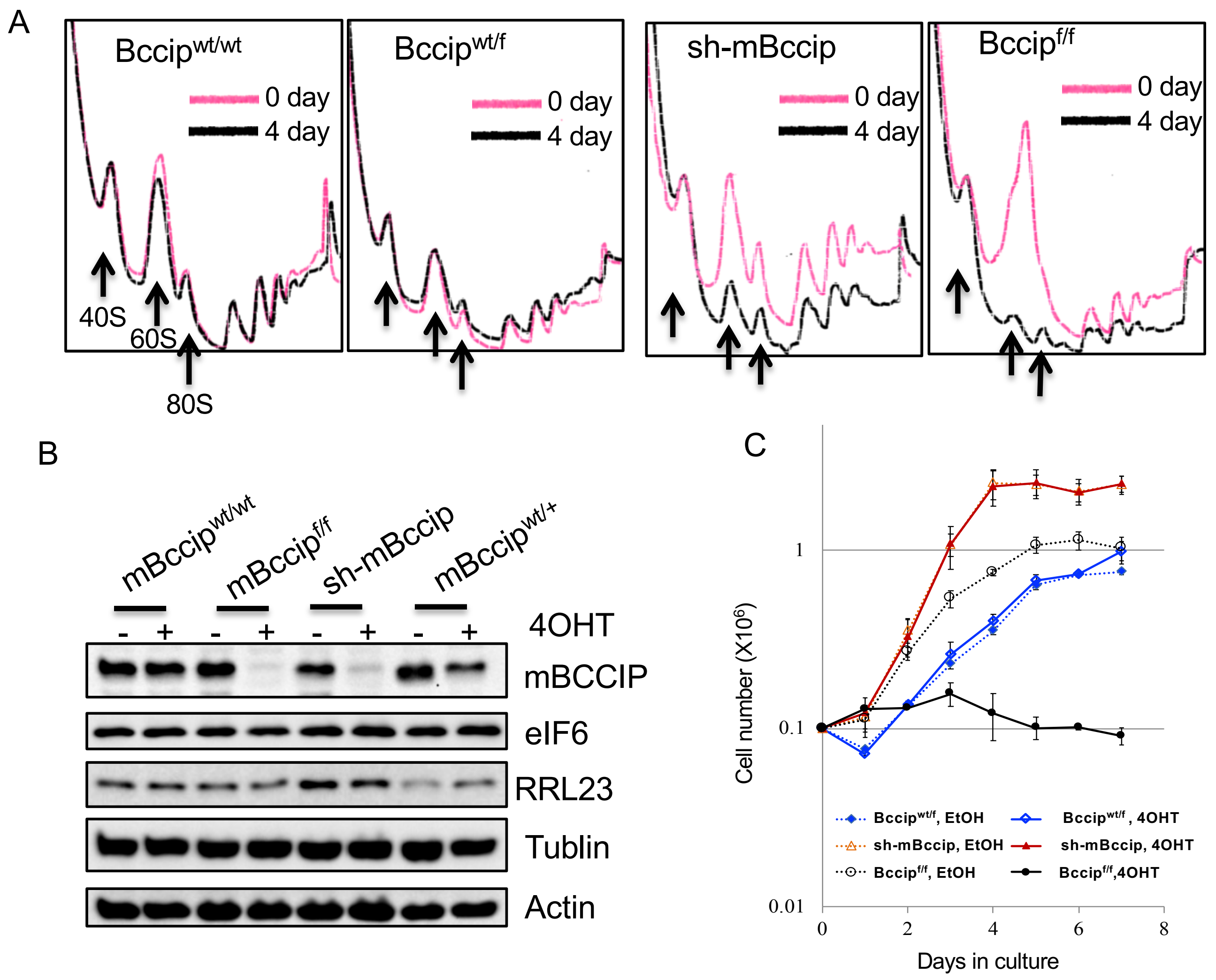

Fig S7. The severity of 60S ribosome biogenesis defect was BCCIP dosage dependent. MEFs with 4OHT-inducible homozygous Bccip knockout ($Bccip^{f/f}$) , heterozygous Bccip knockout ($Bccip^{wt/f}$), shRNA mediated knockdown (sh-mBccip), and $Bccip^{wt/wt}$ were treated with 4OHT for 4 days. Then the MEFs were collected for ribosome profile assay (A) and western blots (B). The growth curves of these cells are shown in panel C.

Fig S8

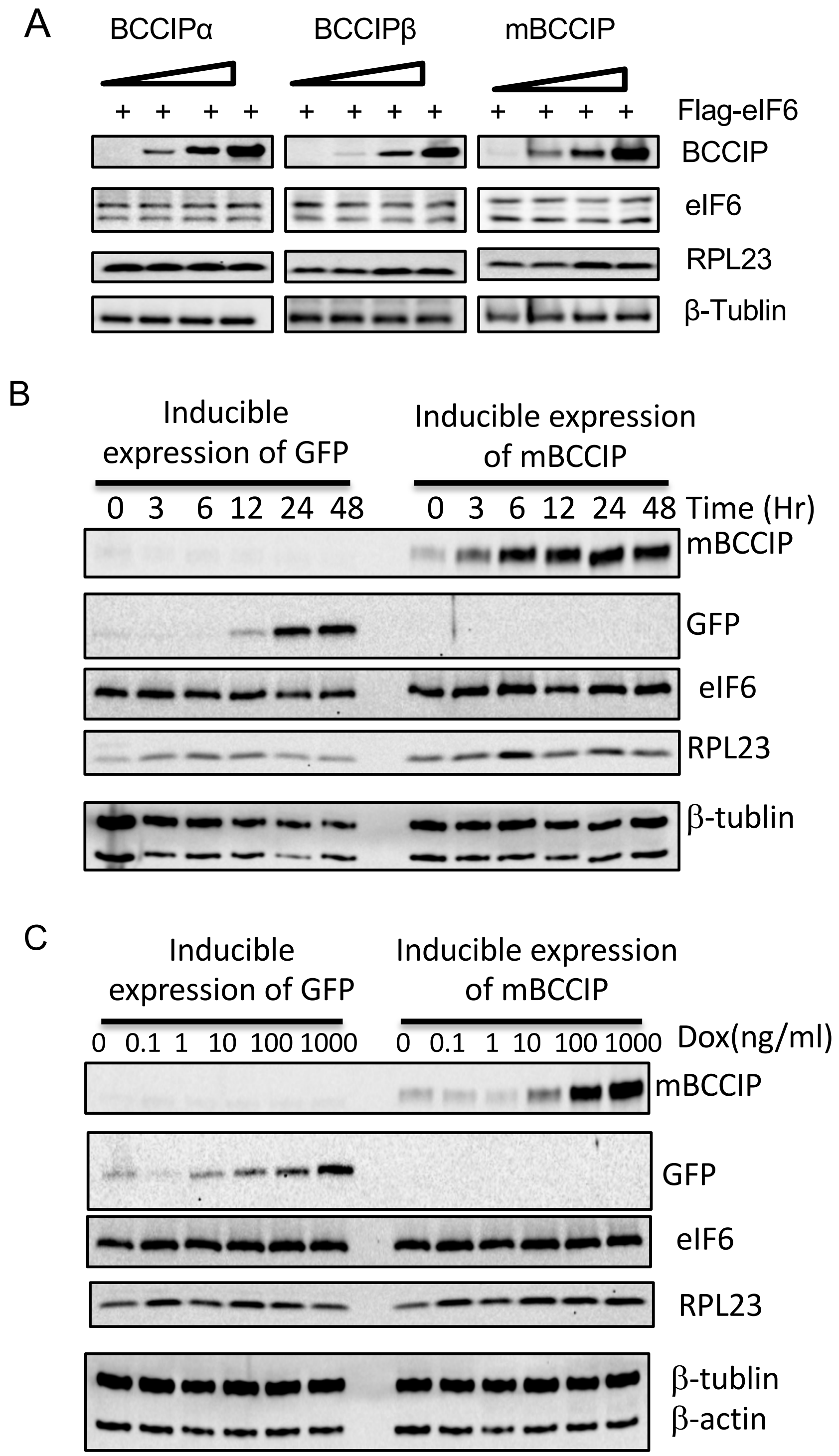

Fig S8. Over-expression of BCCIP did not stabilize eIF6.

(A): Different amount of human BCCIPα, BCCIPβ, and mouse BCCIP expressing plasmids were transient transfected to HEK293 cells with same amount of eIF6 plasmids. Empty of vector was added to reach the same total amount of 3 μg plasmids. After 56 hours, the cells were collected for western blots to detect both endogenous and exogenous eIF6.

(B, C): MEFs with Dox-inducible expression of GFP or mouse Bccip were treated with 200 ng/ml for the indicated lengths of time (B), or with different concentration of Dox for 48 hours (C). The cells were collected for western blots to detect eIF6.

Fig S9

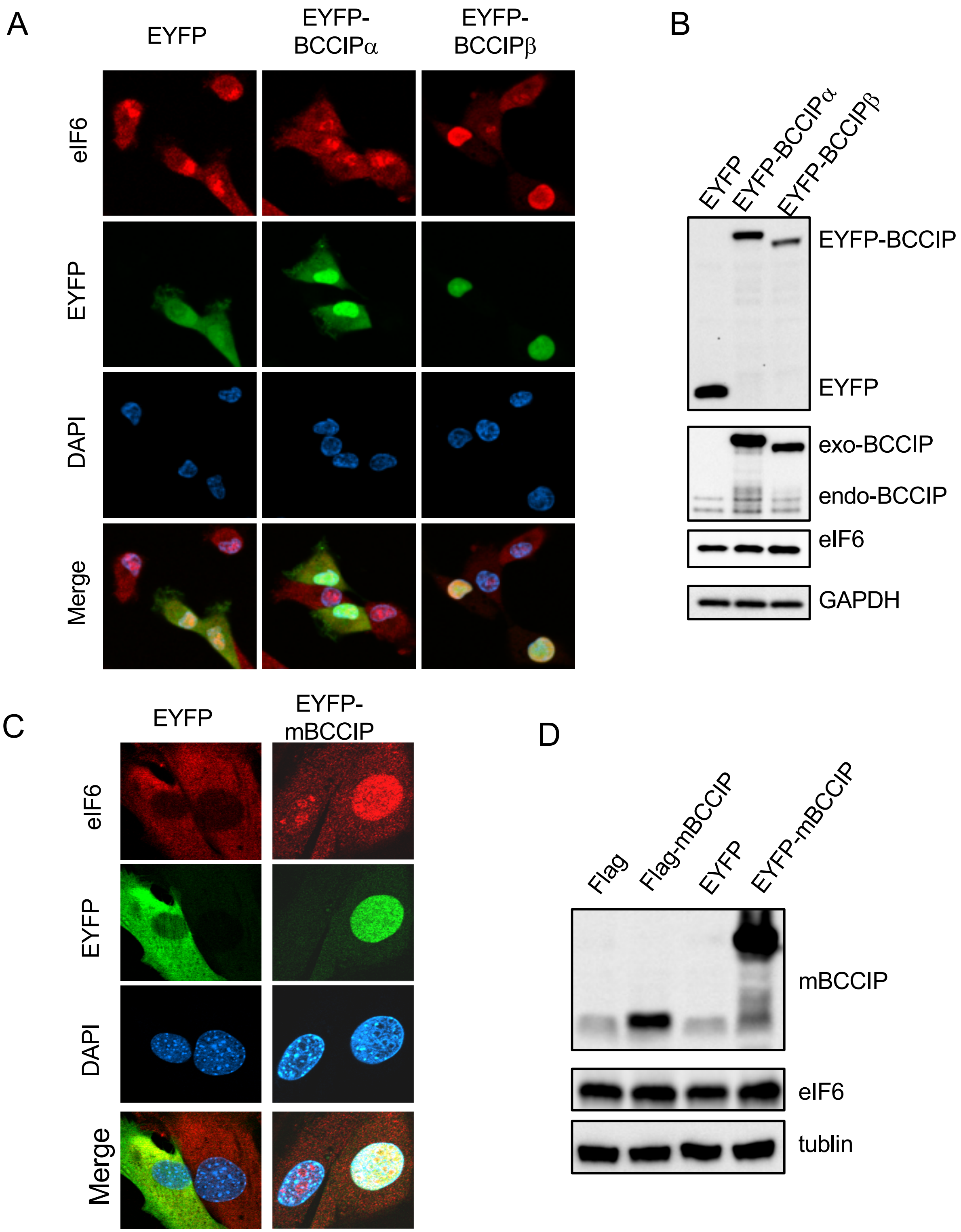

Fig S9. Expression of BCCIPβ and mouse BCCIP, but not BCCIPα, enhanced nuclear localization of eIF6. EYFP-tagged human BCCIPα, BCCIPβ, and mouse BCCIP were transient expressed in HT1080 (A, B) or MEF cells (C, D). The eIF6 in these cells were stained (red) and visualized. Panels A and C are representative images illustrating the eIF6 intensity in the transfected cells (green) and non-transfected (non-green) cells. The expression of the EYFP and EYFP-fusion proteins were verified in panels B and D.

Fig S10

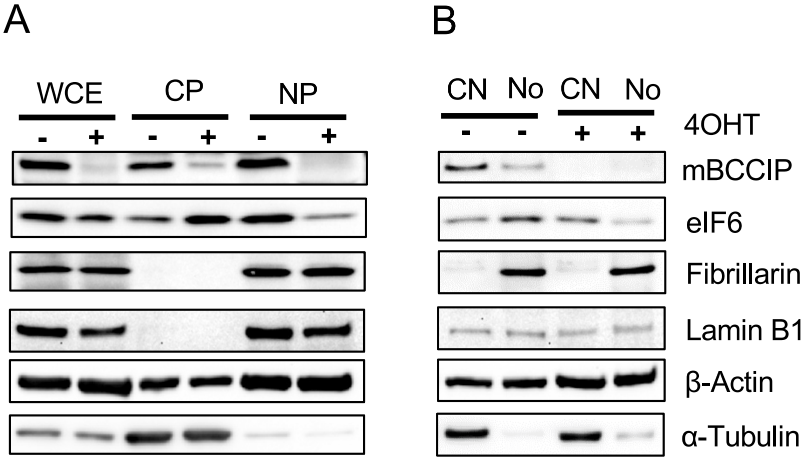

Fig. S10. ***Bccip*** **deletion reduces the nuclear and nucleolus recruitment of eIF6.** The *Bccip*$^{f/f}$*;Rosa-CreERT2*$^{+}$ MEFs were treated with 4OHT to induce *Bccip* deletion, and then collected for two parallel fractionation analyses with Cell Fractionation kit (Cell Signaling Technology #9038) and sucrose gradient centrifugation. In the first fractionation (penel A), the cytoplasm (CP) and nuclear (NP) fractions were collected. In the second fractionation (panel B), the nucleolus (No) and remaining non-nucleolus components including cytoplasmic and nuclear plasma fractions (CN) were collected. These fractions along with the whole cell extract (WCE) were blotted against the indicated proteins.

Fig S11

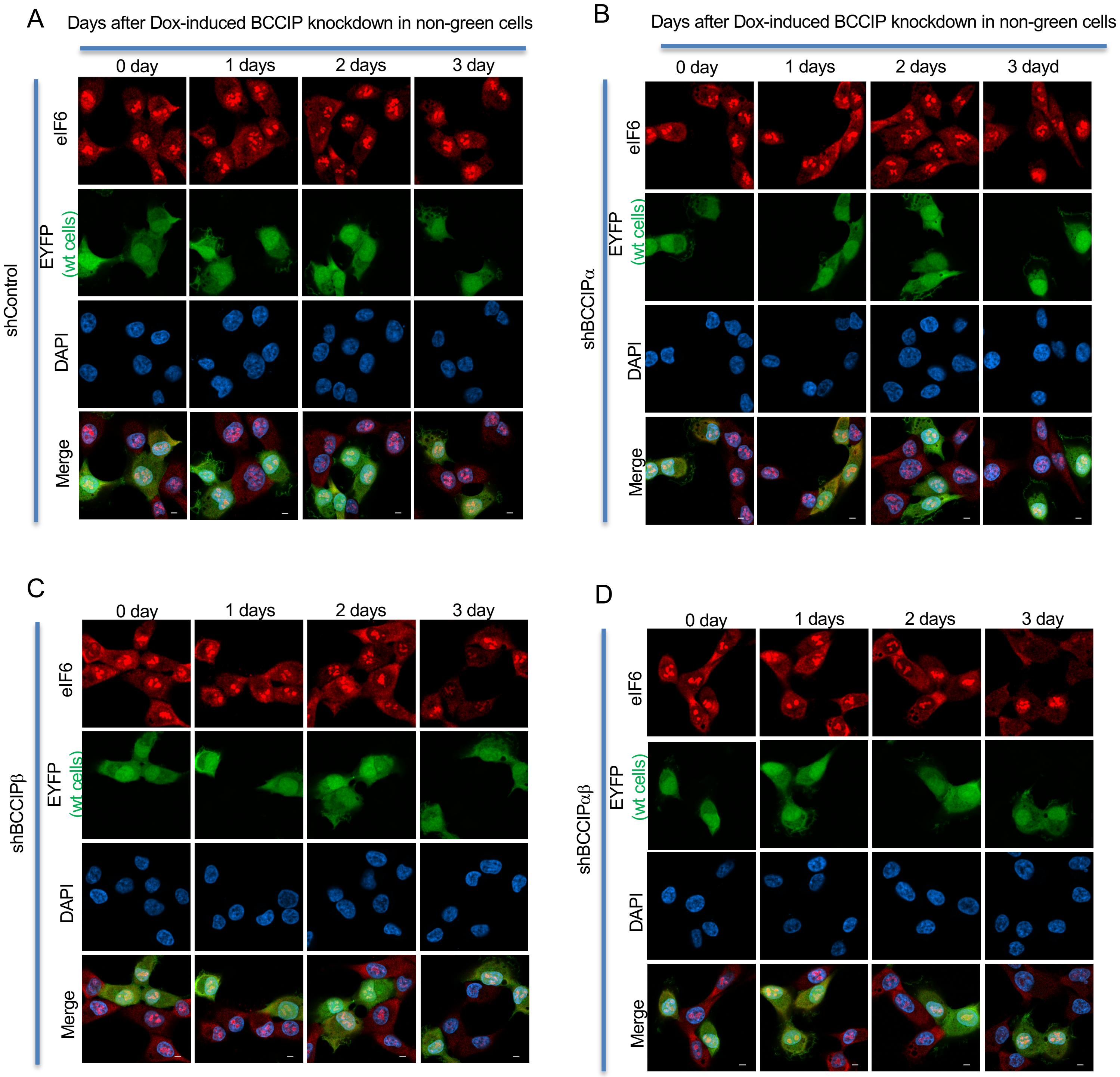

Fig S11. Knockdown of human BCCIPβ, but not BCCIPα, abrogated eIF6 recruitment in the nucleolus. HT1080 cells expressing EYFP were mixed with cell lines that were capable of doxycycline-inducible expressions of control shRNA (shControl, A), BCCIPα specific shRNA (shBCCIPα, B), BCCIPβ specific shRNA (shBCCIPβ, C), and shRNA against both BCCIPα and BCCIPβ isoforms (shBCCIPαβ, D). After the cells were exposed to doxycycline for the indicated length of times, the cells were stained for eIF6 (red). By comparing the eIF6 signal intensity between the green cells (wild type HT1080) and non-green cells that express the indicated shRNA, it can be determined whether the expression of the indicated shRNA in the non-green cells had an effect on the eIF6 intra-cellular distribution. As shown here, the nucleolus localization of eIF6 is dependent on BCCIPβ, but not BCCIPα.

Fig S12

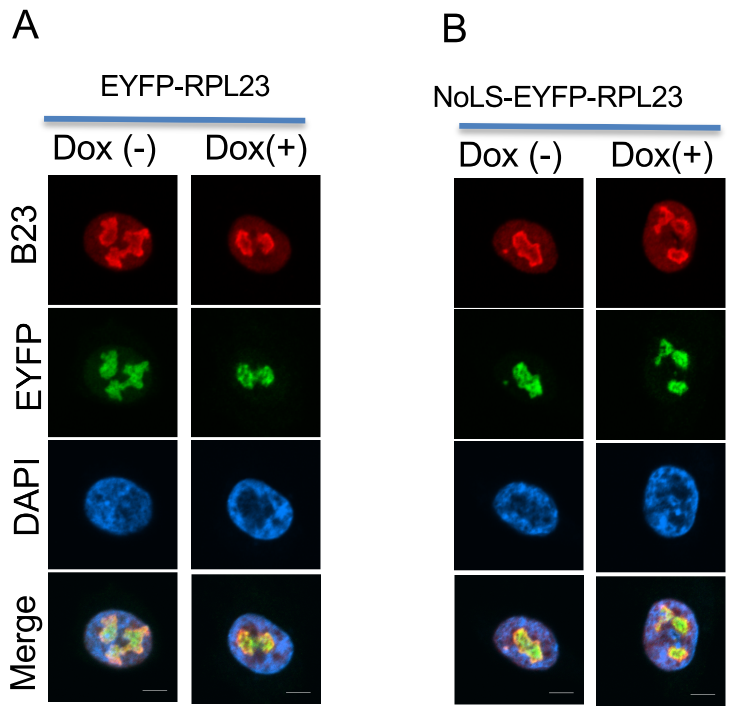

Fig S12. Knockdown of BCCIP did not influence RPL23 nucleolus localization. EYFP-RPL23 (A), or nucleolus localizing sequences (NoLS) fused EYFP-RPL23 (B) were expressed in HT1080 cells capable of Dox-induced BCCIP knock down cells. The cells were cultured with Dox for 3 days to deplete endogenous BCCIPαβ, and re-plated. The next day, the cells were fixed with 4% PFA, and stained with B23. As shown here, the nucleolus localization of RPL23 was not affected by BCCIP depletion in either condition.

Fig S13

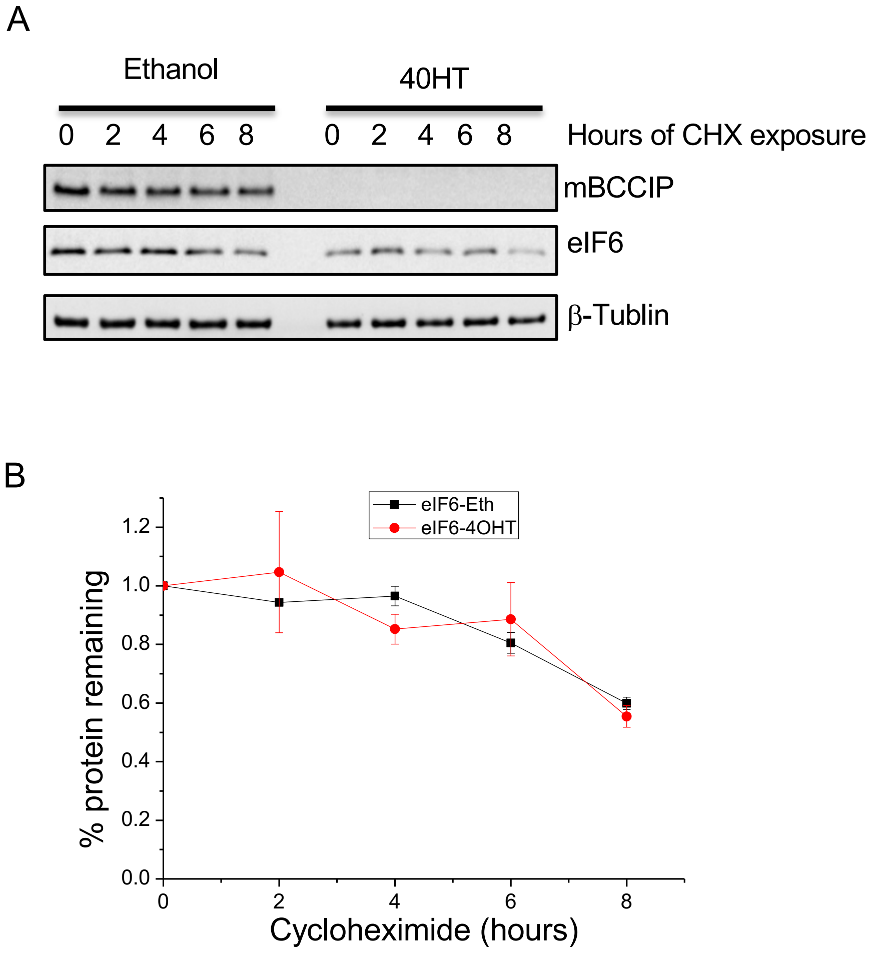

Fig S13. *BCCIP* depletion did not affect the overall stability of the eIF6 proteins. *Bccip$^{f/f}$;Rosa-CreERT2 MEF* were treated with 4OHT to induce *Bccip* deletion for 4 days. Then cycloheximide was added to inhibit new protein synthesis. The relative level of eIF6 was measured by western blots (A) and the quantification is shown in panel B.

Fig S14

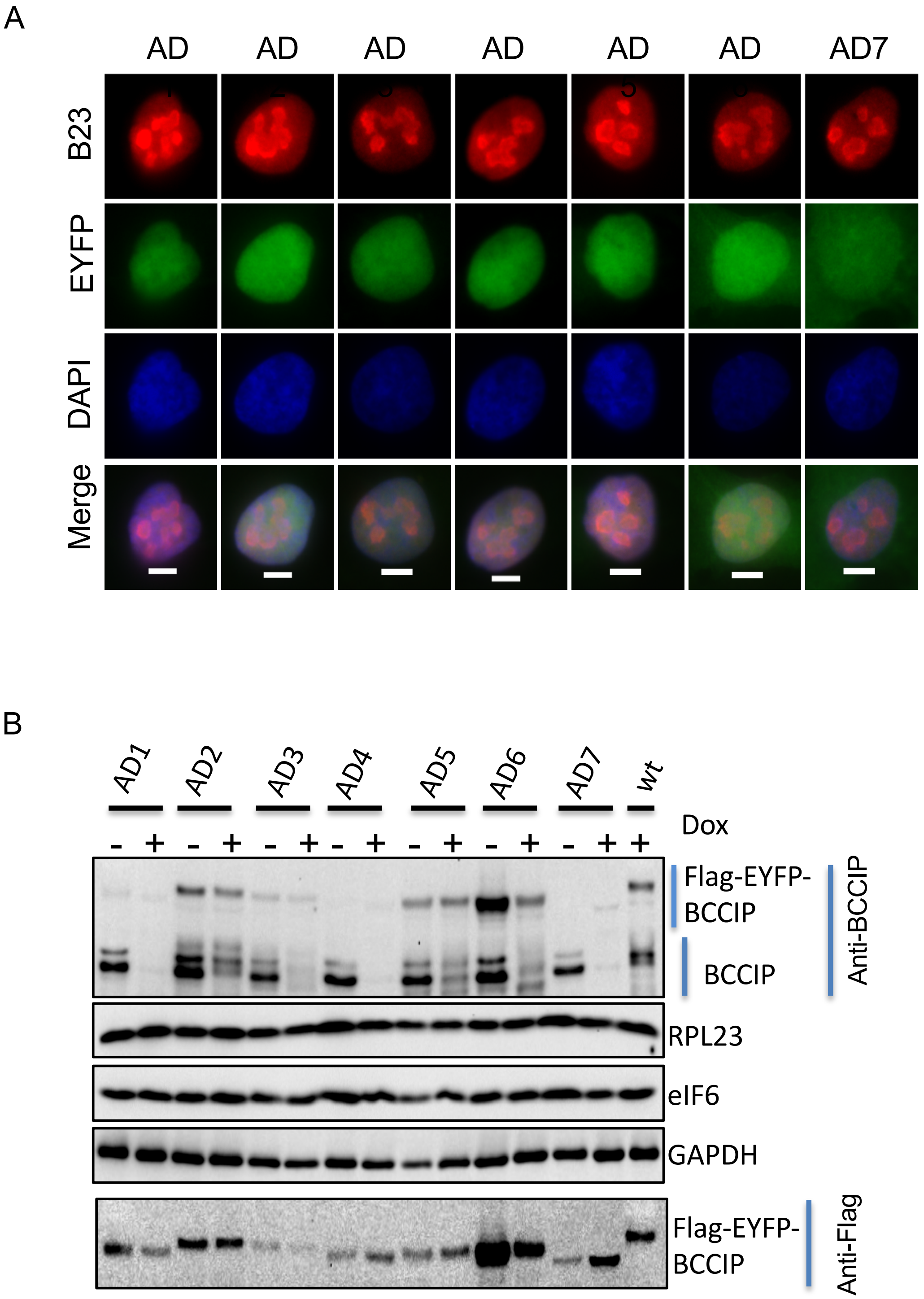

Fig S14. Acidic domain mutants did not influence B23 distribution. The shBCCIP-resistant Flag-EYFP tagged AD mutants were expressed in HT1080 cells capable of Dox-inducible BCCIP knockdown (Fig. 2D). After 3 days of Dox-treatment to deplete the endogenous BCCIP, the cells were fixed with 4% PFA and then stained with B23 (A), the cell extract was analyzed by western blots using the indicated antibodies (B).

Fig S15

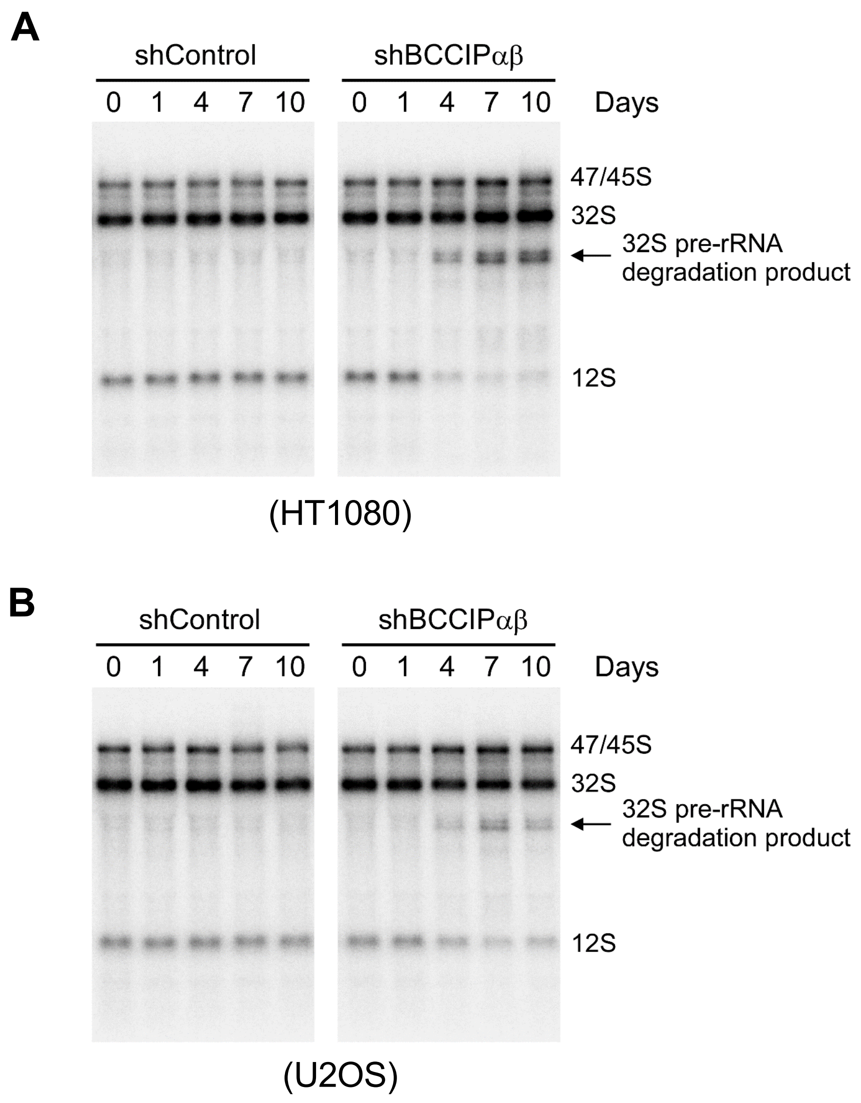

Fig S15. BCCIP depletion resulted in the reduction of 12S pre-rRNA and appearance of pre-rRNA degradation products. HT1080 cells (A) or U2OS cells (B) capable of Dox-inducible BCCIP knock down were treated with Dox for the indicated time. The cells were collected and their RNA were analyzed by Northern hybridizations with probe p4 (Fig 7C).

Fig. S16

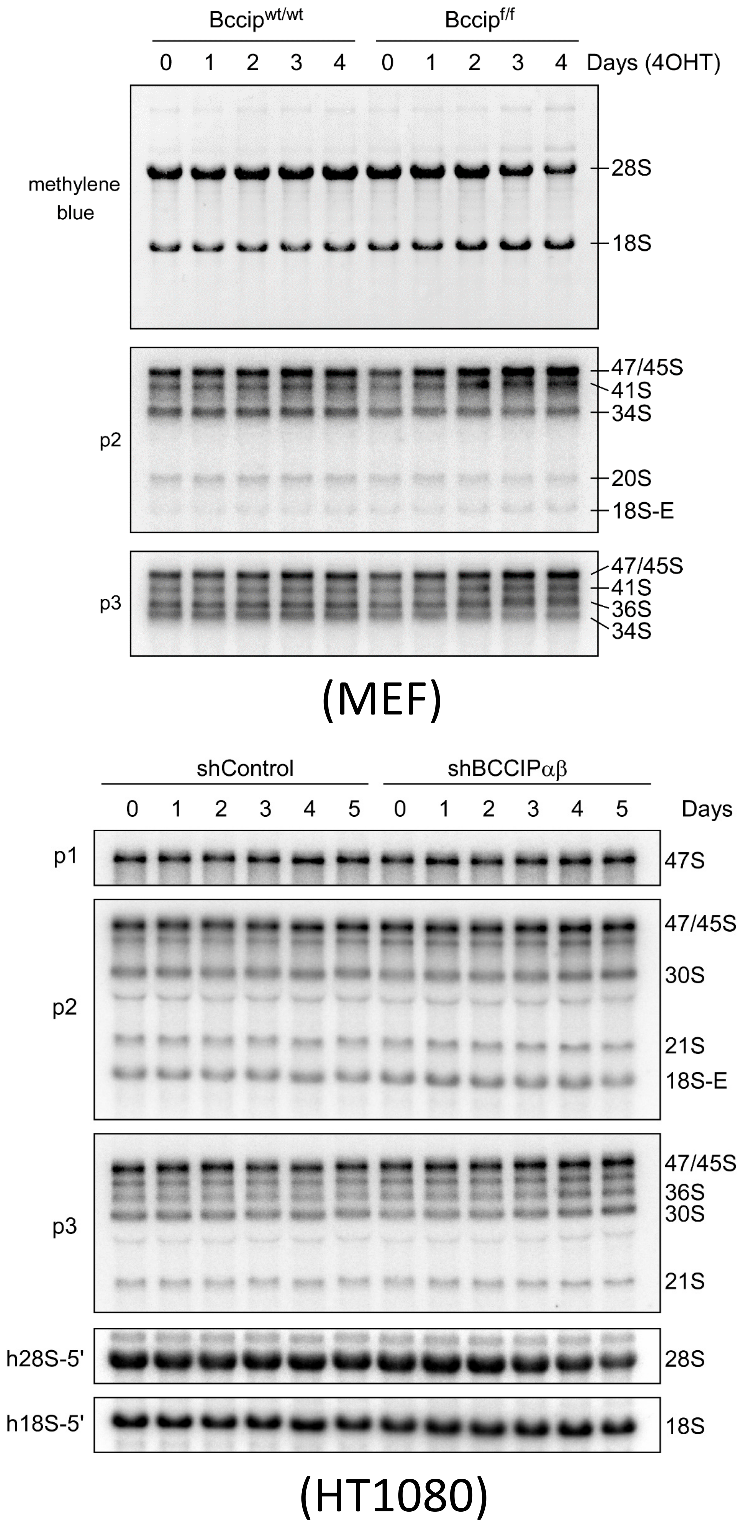

Fig. S16. Detection of rRNA precursors by Northern blots with additional probes (shown on the left) with total RNA isolated from MEF and HT1080 cells. See Fig 7C and Table S1 for probe information.
