# Supplementary material for: BCCIP is required for nucleolar recruitment of eIF6 and 12S pre-rRNA production during 60S ribosome biogenesis
Source: Nucleic Acids Res. 2020 Nov 27;48(22):12817–32. doi: 10.1093/nar/gkaa1114 (PMC7736804; doi:10.1093/nar/gkaa1114)
Supplement: gkaa1114_Supplemental_Files [file gkaa1114_supplemental_files.zip › NAR-00644-R-2020 TABLE S1.pdf]

Table S1. Probes used for rRNA northern blots

Species	Reference pre-rRNA sequence	Probe	Region of complementarity on pre-rRNA, nt	Sequence
Human	U13369.1	p1	10..29,<br>133..152	GACAGGTCGCCAGAGGACAG,<br>GACGCGCGAGAGAACAGCAG
		p2	5520..5548	GCCTCGCCCTCCGGGCTCCGTTAATGATC
		p3	5695..5711	GTCTCCCTCCCGAGTTC
		p4	6870..6884	GGGTCTGCGCTTAGG
		h18S-5'	3736..3754	GAGCCATTCGCAGTTTCAC
		h28S-5'	7945..7961	GGTCGCCACGTCTGATC
Mouse	BK000964.3	p2	5869..5895	TCCTCCACAGTCTCCCGTTTAATGATC
		p3	5975..6008	TTCTCTCACCTCACTCCAGACACCTCGCTCCACA
		p4	7036..7065	ACCCACCGCAGCGGGTGACGCGATTGATCG
		m18S-5'	4061..4080	GGCCGTGCGTACTTAGACAT
		m28S-5'	8132..8149	GGTCGCCACGTCTGATCT
